# Patients and mice with deficiency in the SNARE protein SYNTAXIN-11 have a secondary B cell defect

**DOI:** 10.1084/jem.20221122

**Published:** 2024-05-09

**Authors:** Tamara Kögl, Hsin-Fang Chang, Julian Staniek, Samuel C.C. Chiang, Gudrun Thoulass, Jessica Lao, Kristoffer Weißert, Viviane Dettmer-Monaco, Kerstin Geiger, Paul T. Manna, Vivien Beziat, Mana Momenilandi, Szu-Min Tu, Selina J. Keppler, Varsha Pattu, Philipp Wolf, Laurence Kupferschmid, Stefan Tholen, Laura E. Covill, Karolina Ebert, Tobias Straub, Miriam Groß, Ruth Gather, Helena Engel, Ulrich Salzer, Christoph Schell, Sarah Maier, Kai Lehmberg, Tatjana I. Cornu, Hanspeter Pircher, Mohammad Shahrooei, Nima Parvaneh, Roland Elling, Marta Rizzi, Yenan T. Bryceson, Stephan Ehl, Peter Aichele, Sandra Ammann

**Affiliations:** 1 Institute for Immunology, Center for Microbiology and Hygiene, Medical Center—University of Freiburg, Freiburg, Germany; 2 Cellular Neurophysiology, Center for Integrative Physiology and Molecular Medicine, Saarland University, Homburg, Germany; 3 Faculty of Medicine, Institute for Immunodeficiency, Medical Center—University of Freiburg, Freiburg, Germany; 4 Faculty of Medicine, Center for Chronic Immunodeficiency, Medical Center—University of Freiburg, Freiburg, Germany; 5 Division of Bone Marrow Transplantation and Immune Deficiency, and Department of Pediatrics, Cincinnati Children’s Hospital Medical Center, University of Cincinnati, Cincinnati, OH, USA; 6 Department of Medicine, Center for Hematology and Regenerative Medicine Huddinge, Karolinska Institute, Karolinska University Hospital Huddinge, Stockholm, Sweden; 7 Faculty of Biology, Albert-Ludwigs-University of Freiburg, Freiburg, Germany; 8 Faculty of Medicine, Institute for Transfusion Medicine and Gene Therapy—University of Freiburg, Freiburg, Germany; 9 Department of Neuroscience and Physiology, University of Gothenburg, Gothenburg, Sweden; 10 Laboratory of Human Genetics of Infectious Diseases, Necker Branch, INSERM, Necker Hospital for Sick Children, Paris, France; 11 Imagine Institute, University of Paris-Cité, Paris, France; 12 St. Giles Laboratory of Human Genetics of Infectious Diseases, Rockefeller Branch, The Rockefeller University, New York, NY, USA; 13 Division of Rheumatology and Immunology, Medical University of Graz, Graz, Austria; 14 Department of Urology, Faculty of Medicine, Medical Center—University of Freiburg, Freiburg, Germany; 15 Institute of Medical Microbiology and Hygiene, University Medical Center, Freiburg, Germany; 16 Department of Pathology, Institute of Surgical Pathology, University Medical Center, University of Freiburg, Freiburg, Germany; 17 Division of Pediatric Stem Cell Transplantation and Immunology, University Medical Center Hamburg-Eppendorf, Hamburg, Germany; 18 Department of Microbiology, Immunology, and Transplantation, Clinical and Diagnostic Immunology, KU Leuven, Leuven, Belgium; 19 Dr. Shahrooei Laboratory, Tehran University of Medical Sciences, Tehran, Iran; 20 Division of Allergy and Clinical Immunology, Department of Pediatrics, Tehran University of Medical Sciences, Tehran, Iran; 21 Research Center for Immunodeficiencies, Tehran University of Medical Sciences, Tehran, Iran; 22 Faculty for Medicine, Center for Pediatrics and Adolescent Medicine, Medical Center—University of Freiburg, Freiburg, Germany; 23 Department of Rheumatology and Clinical Immunology, Faculty of Medicine, Medical Center— University of Freiburg, Freiburg, Germany; 24 Division of Clinical and Experimental Immunology, Institute of Immunology, Center for Pathophysiology, Infectiology and Immunology, Medical University of Vienna, Vienna, Austria; 25 Centre for Integrative Biological Signalling Studies, University of Freiburg, Freiburg, Germany; 26 Faculty of Medicine, Clinical Immunology, Medical Center—University of Freiburg, Freiburg, Germany; 27 Division of Clinical Immunology and Transfusion Medicine, Karolinska University Hospital Huddinge, Stockholm, Sweden; 28 Broegelmann Laboratory, Department of Clinical Sciences, University of Bergen, Bergen, Norway

## Abstract

SYNTAXIN-11 (STX11) is a SNARE protein that mediates the fusion of cytotoxic granules with the plasma membrane at the immunological synapses of CD8 T or NK cells. Autosomal recessive inheritance of deleterious *STX11* variants impairs cytotoxic granule exocytosis, causing familial hemophagocytic lymphohistiocytosis type 4 (FHL-4). In several FHL-4 patients, we also observed hypogammaglobulinemia, elevated frequencies of naive B cells, and increased double-negative DN2:DN1 B cell ratios, indicating a hitherto unrecognized role of STX11 in humoral immunity. Detailed analysis of *Stx11*-deficient mice revealed impaired CD4 T cell help for B cells, associated with disrupted germinal center formation, reduced isotype class switching, and low antibody avidity. Mechanistically, *Stx11*^−/−^ CD4 T cells exhibit impaired membrane fusion leading to reduced CD107a and CD40L surface mobilization and diminished IL-2 and IL-10 secretion. Our findings highlight a critical role of STX11 in SNARE-mediated membrane trafficking and vesicle exocytosis in CD4 T cells, important for successful CD4 T cell–B cell interactions. Deficiency in STX11 impairs CD4 T cell–dependent B cell differentiation and humoral responses.

## Teaser

CD4 T helper cells use the SNARE protein SYNTAXIN-11 to promote B cell differentiation, germinal center formation, and class switching by facilitating CD40L mobilization and IL-2 and IL-10 secretion. Variable hypogammaglobulinemia is a novel phenotype of human STX11 deficiency.

## Introduction

Inborn errors with impaired humoral immunity are mostly associated with mutations in genes affecting B cell activation, differentiation, or class switch recombination, like Bruton-Tyrosinkinase (BTK), Igα, activation-induced cytidine deaminase (AID), B cell activating factor receptor, or IL-21r (Khan et al., 1995; Gaspar and Conley, 2000; Revy et al., 2000; Ferrari et al., 2001; Kotlarz et al., 2013). Additionally, many combined immunodeficiencies affecting both T and B cell function (like CD40, Wiskott-Aldrich Syndrome protein, DOCK8, or ARPC1B deficiency) result in impaired humoral immunity. Secondary B cell defects are caused by impaired CD4 T cell help (Crotty, 2019; Cubas et al., 2013), including mutations in CD40L (ligand), ICOS (inducible co-stimulator), SLAM-associated protein, or IL-21 (Pietrella et al., 2004; Gilmour et al., 2003; Grimbacher et al., 2003; Warnatz et al., 2006; Kamperschroer et al., 2006, 2008; Schwartzberg et al., 2009; Salzer et al., 2014), leading to diminished B cell responses to T cell–dependent antigens. Impaired CD4 T cell help results in lack of germinal centers (GCs), B cell class-switch recombination (CSR) deficiencies with markedly low IgA and IgG levels (hypogammaglobulinemia), and impaired affinity maturation (Pietrella et al., 2004; Gilmour et al., 2003; Grimbacher et al., 2003; Warnatz et al., 2006; Kamperschroer et al., 2006, 2008; Schwartzberg et al., 2009; Salzer et al., 2014).

Defects in B cell function in the context of gene mutations affecting soluble N-ethylmaleimide-sensitive factor attachment receptor (SNARE) proteins have not been described. SYNTAXIN-11 (STX11) belongs to the family of Qa-SNARE proteins, which facilitate vesicle fusion (Prekeris et al., 2000; Chen and Scheller, 2001; Fasshauer et al., 1998; Stow et al., 2006). STX11 is a membrane-anchored protein that is recruited to the immune synapse in activated CD8 T and natural killer (NK) cells (Halimani et al., 2014; Dieckmann et al., 2015), where it interacts with the accessory SEC1/MUNC-like protein MUNC18-2 (*STXBP2*). This complex facilitates cytotoxic granule fusion with the plasma membrane and release of effector molecules like perforin and granzymes toward target cells (Arneson et al., 2007; Bryceson et al., 2007; D'Orlando et al., 2013; Halimani et al., 2014). Defective STX11 is associated with impaired lymphocyte cytotoxicity (Bryceson et al., 2007; D'Orlando et al., 2013; Rudd et al., 2006; Marsh et al., 2010). Impaired cytotoxicity can arise from various mutations in genes affecting cytotoxic granule biogenesis, trafficking, or exocytosis. These include mutations in the pore-forming effector molecule PERFORIN-1 (Stepp et al., 1999; Feldmann et al., 2002; Goransdotter Ericson et al., 2001; Voskoboinik et al., 2015), in proteins essential for cytotoxic granule maturation (lysosomal trafficking regulator [LYST]) (Barbosa et al., 1996; Nagle et al., 1996), or those involved in transport and exocytosis: MUNC13-4 (Feldmann et al., 2003; zur Stadt et al., 2005), MUNC18-2 (zur Stadt et al., 2009; Cote et al., 2009; Cetica et al., 2010), or RAB27A (Menasche et al., 2000). Autosomal recessive inheritance of mutations in these genes predisposes to the life-threatening hyperinflammatory syndrome hemophagocytic lymphohistiocytosis (HLH) (Henter et al., 1991; Jordan et al., 2004; Terrell and Jordan, 2013). The clinical presentation of primary HLH patients includes fever, hepatosplenomegaly, cytopenia, hemophagocytosis, frequent liver inflammation, and/or neurological manifestations (Ishii et al., 2005; Rudd et al., 2006; Horne et al., 2008; Marsh et al., 2010). The disease usually develops early in life and is fatal if not treated immediately with aggressive immunochemotherapy, followed by hematopoietic stem cell transplantation (Arico et al., 2001; Chandrakasan and Filipovich, 2013; Janka, 2005; Janka and Lehmberg, 2014). Mice with defects in the corresponding genes do not develop HLH spontaneously (Jordan et al., 2004; Crozat et al., 2007; Kogl et al., 2013; Sepulveda et al., 2013; Jessen et al., 2011; Pachlopnik Schmid et al., 2008), but infection with lymphocytic choriomeningitis virus (LCMV) induces the complete clinical manifestation of HLH with CD8 T cells and IFNγ as the main drivers of the pathology (Jordan et al., 2004; Pachlopnik Schmid et al., 2009; Kogl et al., 2013; Rood et al., 2016). To our knowledge, CD4 T and B cells have not been implicated in disease development. Furthermore, a function of the SNARE protein STX11 in these cell populations has—so far—not been reported.

Prompted by sporadic reports of hypogammaglobulinemia in familial HLH type 5 (FHL-5) (Rohr et al., 2010; Meeths et al., 2010; Pagel et al., 2012; Esmaeilzadeh et al., 2015), we measured antibody levels in sera of FHL-2, FHL-3, and FHL-4 patients and analyzed B cell subsets using IgD and CD27. B cell differentiation, GC formation, isotype switching, and antibody avidity were analyzed in the corresponding mouse models. To study the interaction dynamics of *Stx11*-deficient or wildtype (WT) CD4 T cells with B cells, we established an antigen-specific in vitro T/B cell interaction assay and analyzed membrane trafficking, CD107a and CD40L mobilization, and cytokine production and performed a broader screening of proteins in CD4 T cells by mass spectrometry (MS). Our findings collectively reveal that STX11 plays a crucial role in enabling CD4 T cells to properly perform their helper function, particularly in their interaction with B cells.

## Results

### Hypogammaglobulinemia and increased frequencies of naive B cells in FHL-4 patients

**Figure 1.**
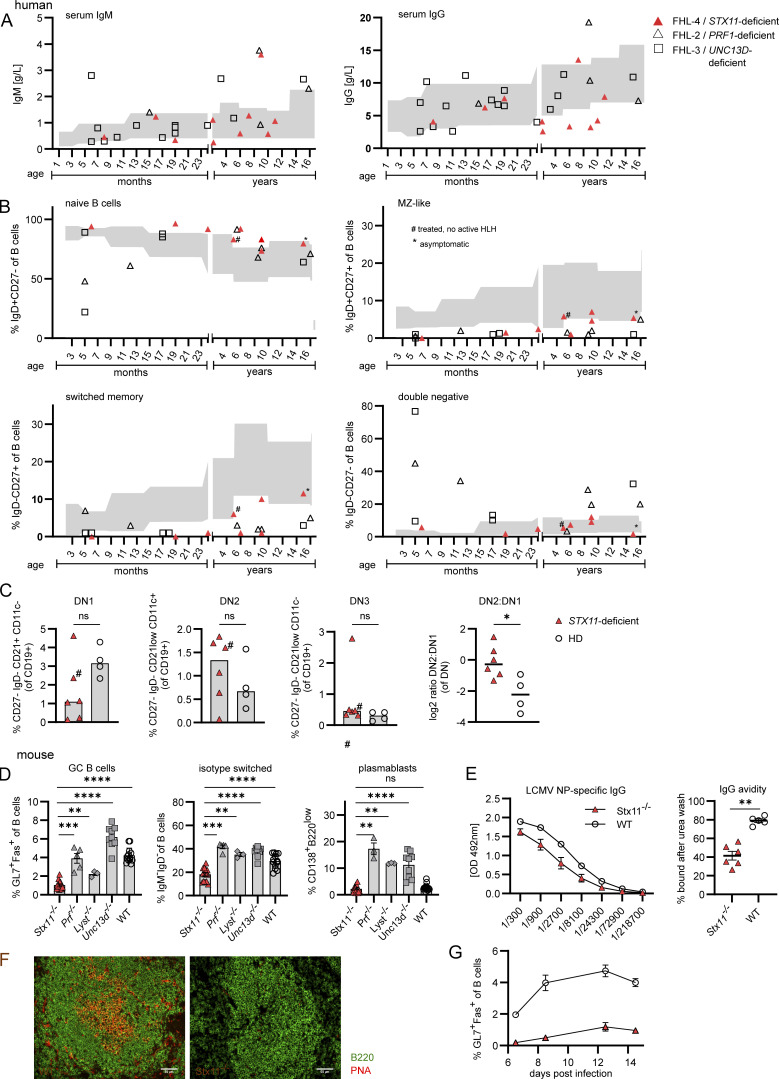
**Hypogammaglobulinemia and impaired GC formation in FHL-4 patients and *Stx11***^***−/−***^ **mice.** Human: **(A and B)** Gray area: age-related reference values (Oster, 2015; Piątosa et al., 2010); # treated; * asymptomatic. **(A)** Sera from 4 FHL-2 (*PRF1*-deficiency), 16 FHL-3 (*UNC13D*-deficiency), and 10 FHL-4 (*STX11*-deficiency) patients with acute HLH, (≥5 mo of age). i.v. Ig–substituted patients were excluded. **(B)** B cell subpopulations in human samples shown as % of total B cells (six FHL-2, five FHL-3, and eight FHL-4 patients): IgD^+^CD27^−^ (naive), IgD^+^CD27^+^ (MZ-like), IgD^−^CD27^+^ (switched memory), and IgD^−^CD27^−^ (DN). **(C)** DN subpopulations of frozen PBMC samples of six FHL-4 patients and four healthy donors (HD). DN1: differentiation via GCs, DN2: extrafollicular differentiation. Mouse: **(D)** Frequency of isotype-switched B cells, GC B cells (Fas^+^GL7^+^), isotype-switched B cells (IgM^−^IgD^−^ of CD19^+^B220^+^), and plasmablasts (CD138^+^B220^low^) in spleens of *Stx11*^*−/−*^ (*n* = 15, five independent experiments), *Prf1*^*−/−*^(*n* = 5, two independent experiments), *Unc13d*^*−/−*^ (*n* = 9, three independent experiments), and *Lyst*^*−/−*^ (*n* = 3, one experiment) and WT (C57BL/6N *n* = 4, *Stx11*^*+/+*^ *n* = 9, five independent experiments) mice d12–14 p.i. with 200 PFU LCMV-WE i.v. **(E)** LCMV-NP–specific IgG in sera of *Stx11*^*−/−*^ and WT mice on d28 p.i. analyzed by ELISA (WT: C57BL/6N *n* = 1 and *Stx11*^*+/+*^ *n* = 4, *Stx11*^*−/−*^ *n* = 7, one experiment). IgG avidity measurements. (WT: C57BL/6N *n* = 1 and *Stx11*^*+/+*^ *n* = 4, *Stx11*^*−/−*^ *n* = 7, one representative experiment). **(F)** Representative spleen sections stained for B220 (green) and PNA (red) (*n* = 4 independent experiments). Scale bar 50 µm. **(G)** *Stx11*^*−/−*^ (*n* = 6–8) and WT littermates (*Stx11*^*+/+*^) (*n* = 4–10) were infected with 200 PFU LCMV i.v. d14 p.i.: GC B cells and their % over time (*n* = 2 independent experiments). **(A–G)** Data are shown as mean and SEMs. Statistical analysis was performed using Mann–Whitney *U* test; *P < 0.05, **P < 0.01, *** P < 0.001, **** < 0.0001, ns indicates not significant.

**Figure S1.**
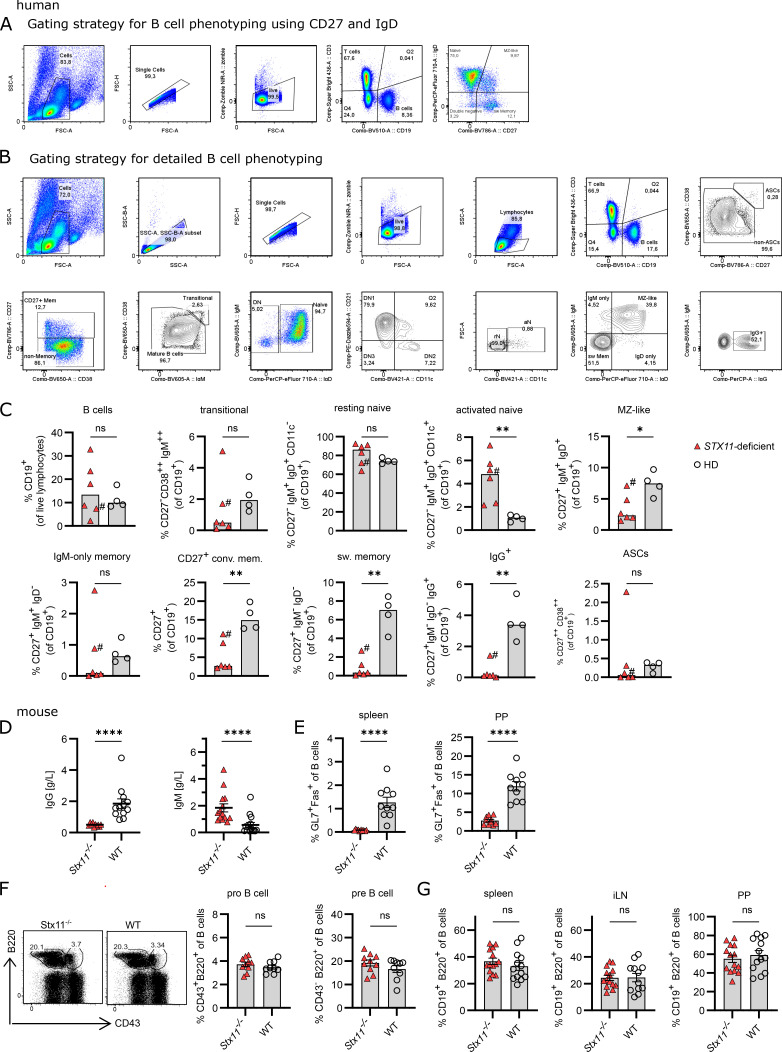
**B cell phenotype of humans and mice with STX11 deficiency.** Human: **(A)** Gating strategy for B cell subpopulation from fresh blood samples or frozen PBMCs related to Fig. 1 B. Naive B cells (CD19^+^IgD^+^CD27^−^), MZ B cells (CD19^+^IgD^+^CD27^+^), DN B cells (CD19^+^IgD^−^CD27^−^), and switched (sw) memory B cells (CD19^+^IgD^−^CD27^+^). **(B)** Gating strategy for detailed B cell phenotyping and DN B cell subsets of peripheral B cells, related to Fig. 1 C and Fig. S1 C. **(C)** Detailed B cell phenotyping of peripheral B cells from frozen PBMCs of six *STX11*^*−/−*^ (FHL-4) patients and four healthy controls. #, treated at the time of analysis. Mouse: **(D)** Serum IgG of *Stx11*^*−/−*^ (*n* = 10) and WT (*Stx11*^*+/+*^ *n* = 12) in *n* = 4 independent experiments and IgM levels in naive mice *Stx11*^*−/−*^ (*n* = 14) or WT (*Stx11*^*+/+*^ *n* = 14 and *Stx11*^*+/−*^ *n* = 2) in *n* = 4 independent experiments. **(E)** GC B cells (GL7^+^Fas^+^ of CD19^+^B220^+^) in spleen and Peyer’s patches (PP) in naive mice (*Stx11*^*−/−*^ *n* = 10) or WT (*Stx11*^*+/+*^ *n* = 9) in *n* = 3 independent experiments. **(F)** Frequencies of pro-B cells (CD43^+^B220^+^) and pre-B cells (CD43^−^B220^+^) in BM *Stx11*^*−/−*^ (*n* = 9) and WT (*Stx11*^*+/+*^ *n* = 9) in *n* = 3 independent experiments. **(G)** B cells (CD19^+^B220^+^) in spleen, inguinal lymphnodes (iLN), and Peyer’s patches (PP) (*Stx11*^*−/−*^ *n* = 13) or WT (*Stx11*^*+/+*^ *n* = 13) in *n* = 4 independent experiments. **(C–G)** Mean and SEMs are shown of Mann–Whitney *U* test, depending on normal distribution; *P < 0.05, **P < 0.01, **** < 0.0001, ns indicates not significant.

Hypogammaglobulinemia has been sporadically reported in patients with hypomorphic mutations in *STXBP2* (FHL-5) and late HLH onset (Rohr et al., 2010; Meeths et al., 2010; Pagel et al., 2012; Esmaeilzadeh et al., 2015). To determine if hypogammaglobulinemia is a feature of other forms of FHL, we analyzed IgM and IgG levels in sera of FHL-2 (*PRF1* deficient), FHL-3 (*UNC13D* deficient), and FHL-4 (*STX11* deficient) patients with acute HLH (patient information: Table S1). To avoid confounding our assessment of IgG levels by maternally transferred antibodies, patients under 5 mo of age were excluded. Similarly, samples from patients receiving Ig therapy were also excluded. IgM levels were predominantly normal for all FHL patients, except one FHL-3 and two FHL-4 patients with lower levels than the age-related reference values (gray area) (Oster, 2015) (Fig. 1 A, left). Five out of 10 (50%) FHL-4 patients had IgG levels lower than the age-related reference values, whereas hypogammaglobulinemia was observed in only two out of 20 (10%) FHL-2/FHL-3 patients (Fig. 1 A, right). B cell subsets of eight FHL-4 and 11 FHL-2/3 patients with active HLH were analyzed with a standard diagnostic staining panel using the markers IgD and CD27 (Fig. S1 A). In five (four with active HLH and one under treatment) of the eight FHL-4 samples, we detected higher frequencies of naive (IgD^+^CD27^+^) B cells compared with the age-related reference values (Piatosa et al., 2010), whereas only one out of 11 FHL-2/3 patients had elevated levels of naive B cells (Fig. 1 B, upper left). The majority of all FHL patients had reduced relative frequencies of marginal zone (MZ)–like B cells (IgD^+^CD27^+^) and switched memory B cells (IgD^−^CD27^+^) (Fig. 1 B, upper right and lower left). Interestingly, during acute HLH, seven out of 11 FHL-2/3 patients had increased frequencies of double negative (DN) B cells (IgD^−^CD27^−^) in comparison with the FHL-4 patients with normal DN B cell frequencies (Fig. 1 B, lower right). Extended B cell phenotyping of six frozen peripheral blood mononuclear cell (PBMC) samples from FHL-4 patients compared with four healthy controls showed normal percentages of B cells, slightly reduced transitional, and slightly increased resting naive B cells (Fig. S1 C). However, in comparison with healthy controls, activated naive B cells were significantly increased in FHL-4 patients, and percentages of MZ, CD27^+^ conventional memory, switched memory, and IgG^+^ B cells were reduced (Fig. S1, B and C). Detailed analysis of DN B cell subpopulations in FHL-4 patients demonstrated decreased DN1 B cells, which primarily develop via GC-dependent pathways, and increased GC-independent DN2 B cells, leading to an increased DN2:DN1 ratio, suggesting an extrafollicular differentiation route (Fig. 1 C). Taken together, FHL-4 patients had normal IgM and mostly reduced IgG levels, increased frequencies of activated naive B cells, reduced memory and isotype-switched memory B cells, and an increased DN2:DN1 ratio, suggesting that B cells of *STX11*-deficient patients were impaired in their GC-dependent differentiation and isotype switching.

### *Stx11* deficiency is associated with impaired GC formation

To decipher the role of STX11 in humoral immunity, additional analyses were performed in the corresponding preclinical HLH mouse model. In *Stx11*^−/−^ mice, we observed strongly reduced IgG and increased IgM levels under steady-state conditions (Fig. S1 D). Furthermore, GC B cells (GL7^+^Fas^+^) were hardly detectable in spleen and Peyer’s patches (Fig. S1 E). To exclude a developmental B cell defect, we analyzed bone marrow (BM) from *Stx11*^−/−^ mice (Hardy et al., 1991). Early B cell development was unaffected with comparable frequencies of pro-B cells (B220^+^CD43^+^) and pre-B cells (B220^+^CD43^−^) in *Stx11*^−/−^ and WT littermates (Fig. S1 F). Similar distribution of mature B cells in secondary lymphoid organs suggested normal peripheral B cell development (Fig. S1 G).

**Figure S2.**
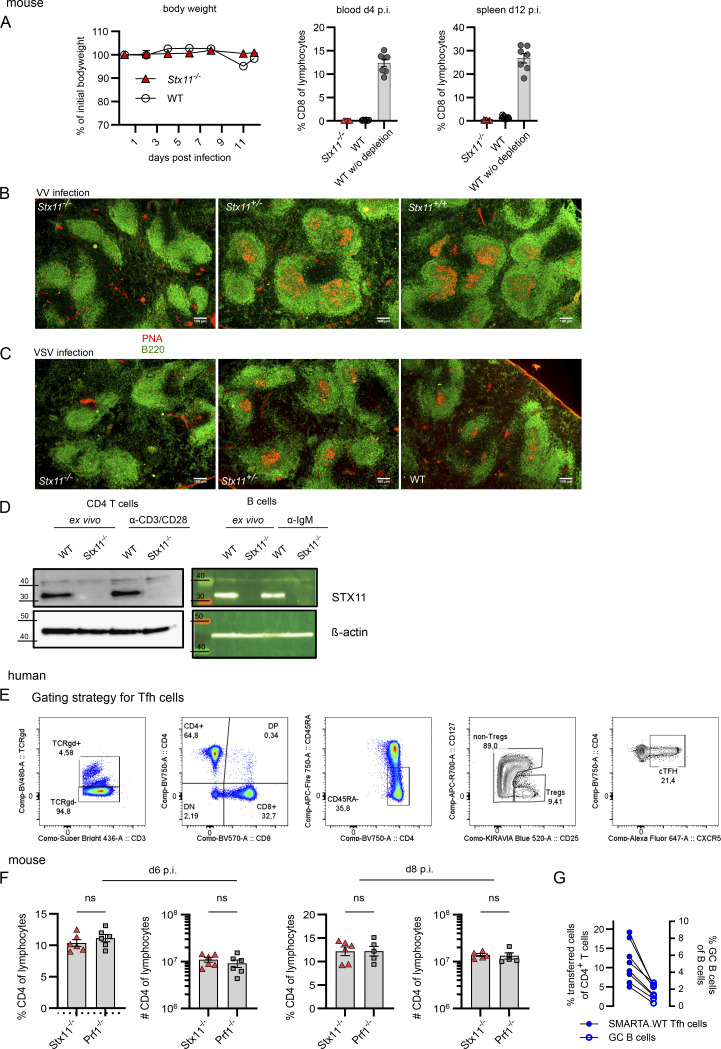
**STX11 in T and B cells.** Mouse: **(A)** Efficiency of CD8 T cell depletion showing body weight over time (left) and frequency of CD8 T cells in the blood d4 p.i. and d12 in the spleen p.i. Related to Fig. 2 A. **(B and C)** Representative spleen sections d14 p.i. with VV (B) or VSV (C) in *Stx11*^*−/−*^, *Stx11*^*+/−*^, and *Stx11*^*+/+*^ mice. Scale bar 100 µm, B220 (B cell area) in green and PNA (GC-B cells) in red. **(D)** Western blot (5 µg total protein) of STX11 expression in naive and stimulated purified splenic CD4 T cells and B cells from WT (*Stx11*^*+/+*^) and *Stx11*^*−/−*^ mice. Molecular mass is shown in kilodaltons. Human: **(E)** Gating strategy for Tfh cells; related to Fig. 3 A. Mouse: **(F)** CD4 T cell percentages and frequencies in spleen d6 (*Stx11*^*−/−*^ *n* = 6, *Prf1*^*−/−*^ *n* = 6) and d8 (*Stx11*^*−/−*^ *n* = 6, *Prf1*^*−/−*^ *n* = 5) p.i. with 200 PFU LCMV; each *n* = 2 independent experiments. **(G)** Correlation between frequencies of transferred SMARTA.WT into *Stx11*^*−/−*^ (*n* = 8) mice and GC B cell frequencies after d12 p.i. with 200 PFU LCMV of *n* = 2 independent experiments. **(A–G)** Mean and SEMs are shown of Mann–Whitney *U* test, depending on normal distribution; ns indicates not significant. Source data are available for this figure: SourceData FS2.

**Figure 2.**
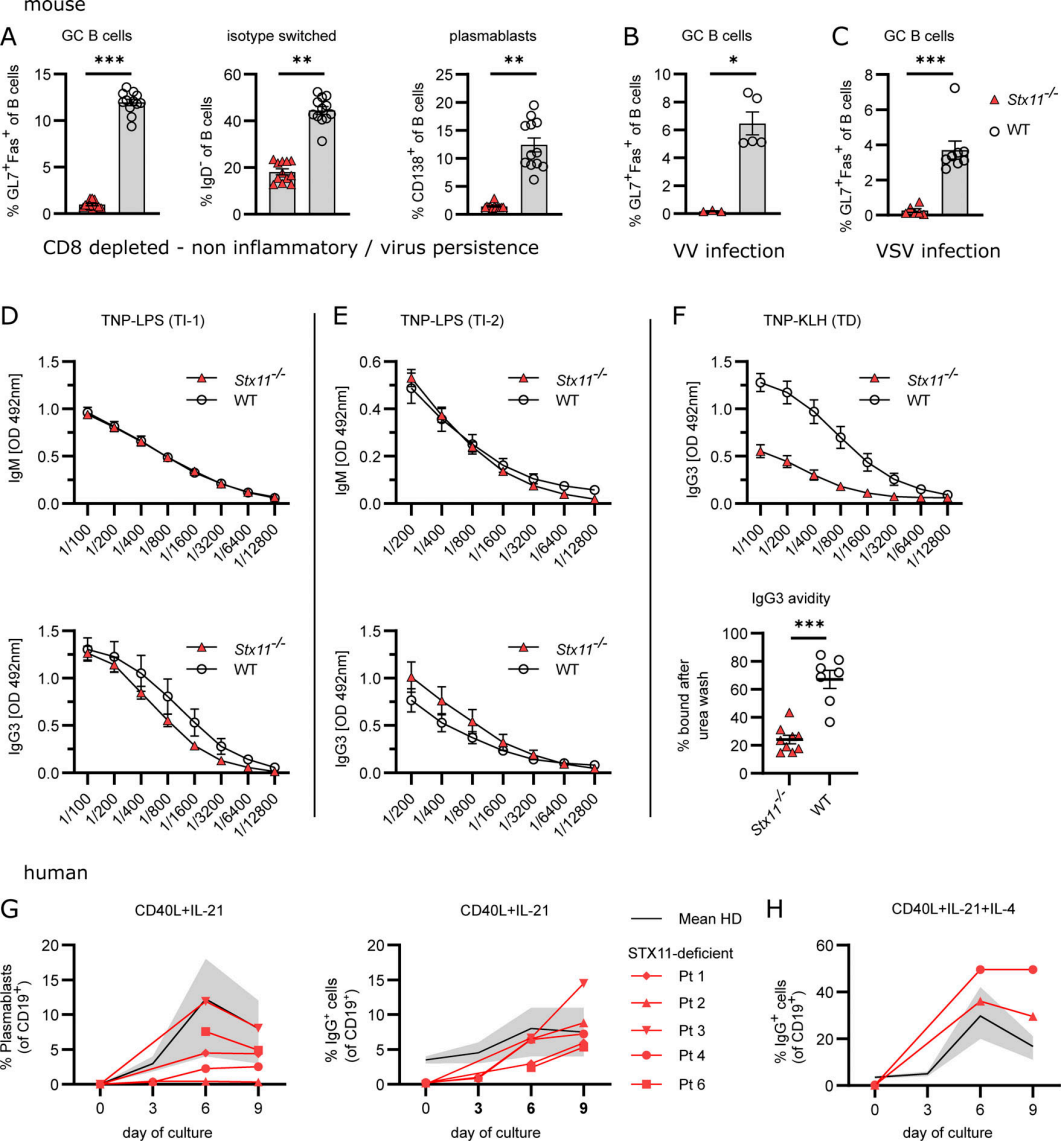
**Impaired B cell responses toward TD antigens in *Stx11***^***−/−***^ **mice.** Mouse: **(A)** Analysis of B cell differentiation in spleens of CD8-depleted (day −3 and −1) *Stx11*^*−/−*^ (*n* = 12) and WT mice (*n* = 12: *Stx11*^*+/+*^ *n* = 6 and C57BL/6N *n* = 6) d12 p.i. with 200 PFU LCMV-WE (*n* = 3 independent experiments). **(B)** *Stx11*^*−/−*^ (*n* = 3) and WT (*n* = 6: *Stx11*^*+/+*^ *n* = 1, *Stx11*^*+/−*^ *n* = 2 and C57BL/6N *n* = 2) infected with 2 × 10^6^ PFU VV i.p.; GC B cells (Fas^+^GL7^+^CD19^+^B220^+^) in spleen (*n* = 1 experiment). **(C)** *Stx11*^*−/−*^ (*n* = 6) and WT (*n* = 8: *Stx11*^*+/+*^ *n* = 1, *Stx11*^*+/−*^ *n* = 3, and C57BL/6N *n* = 4) infected with 2 × 10^6^ PFU VSV i.v.; GC B cells (Fas^+^GL7^+^CD19^+^B220^+^) in spleen (*n* = 2 independent experiments). **(D and E)** IgM (top) and IgG3 (bottom) antibody responses 1 wk after TI immunization with (D) 50 µg TNP-0.5-LPS i.p. *Stx11*^*−/−*^ (*n* = 9) and WT (C57BL/6N *n* = 6, *Stx11*^*+/+*^ *n* = 1) (*n* = 2 independent experiments) or (E) 50 µg TNP-Ficoll i.p. *Stx11*^*−/−*^ (*n* = 7) and WT (*Stx11*^*+/+*^ *n* = 6) (*n* = 2 independent experiments). **(F)** *Stx11*^*−/−*^ (*n* = 9) and WT (C57BL/6N *n* = 3, *Stx11*^*+/+*^ *n* = 4) mice immunized with 100 µg TNP-KLH in Sigma-Aldrich Adjuvants System i.p. and 21 days later boosted with 50 µg TNP-KLH in PBS. Serum levels of IgG3 antibodies were measured 1 wk after boost (day 28 after primary immunization) (*n* = 2 independent experiments) (top). IgG3 avidity measurements (day 28 after primary immunization) (bottom). Relative avidity is reflected by calculation of the area under the curve (AUC) of the titration curve with 8 M urea or and with PBS treatment, respectively (% AUC = AUC+urea/AUC+PBS*100) *Stx11*^*−/−*^ (*n* = 9) and WT (C57BL/6N *n* = 3, *Stx11*^*+/+*^ *n* = 4) (*n* = 2 independent experiments). **(G and H)** Purified naive human B cells were stimulated with (G) CD40L+IL-21 or (H) CD40L+IL-21+IL-4. Plasmablasts (CD19^+^CD38^+^CD27^+^) and isotype-switched IgG^+^CD19^+^ B cell differentiation is shown. The gray area shows the normal range of four healthy donors with mean (black line). **(A–H)** Mean and ± SEMs of Mann–Whitney *U* test are shown; *P < 0.05, **P < 0.01, *** P < 0.001, ns indicates not significant.

Since most patient samples were obtained in the context of HLH, we wanted to exclude effects of hyperinflammation and IFNγ-driven extrafollicular B cell differentiation. For this, B cell responses were analyzed in LCMV-infected HLH-prone mice and WT mice. *Prf1*^*−/−*^, *Unc13d*^*−/−*^, and *Lyst*^*−/−*^ mice showed higher frequencies of GC B cells, isotype-switched B cells, and plasmablasts (CD138^+^B220^low^) compared with *Stx11*^−/−^ mice (Fig. 1 D). This indicates that impaired humoral immunity in *Stx11*^*−/−*^ mice is not a direct consequence of HLH-associated hyperinflammation. Further analysis of humoral immunity demonstrated decreased levels of LCMV-nucleoprotein (NP)–specific IgG antibodies, along with significantly lower avidity in sera of *Stx11*^−/−^ mice (Fig. 1 E). Immunohistological analysis confirmed a lack of GCs in spleen sections of *Stx11*^−/−^ mice (Fig. 1 F), coupled with a lower frequency of GC B cells already by day (d) 6 post infection (p.i.) (Fig. 1 G), suggesting impaired GC formation. To further test the hypothesis that the B cell phenotype is not a consequence of hyperinflammation, we depleted WT and *Stx11*^−/−^ mice of CD8 T cells to analyze B cell differentiation under non-inflammatory conditions (no body weight loss) after LCMV infection (Fig. S2 A). *Stx11*^−/−^ mice still showed significantly lower frequencies of GC B cells, isotype-switched B cells, and plasmablasts (Fig. 2 A), demonstrating that the defect in peripheral B cell maturation is not the result of acute hyperinflammation or CD8 T cell/IFNγ-driven immunopathology. Together with the increased DN2:DN1 ratio seen in human PBMC samples and the low frequency of isotype-switched B cells, B cell differentiation in STX11 deficiency is suggested to mainly occur extrafollicularly, independent of GC formation (Cerutti, 2008; Roco et al., 2019). Importantly, further viral infections with vaccinia virus (VV) or vesicular stomatitis virus (VSV) confirmed the impaired GC formation (Fig. S2, B and C) and reduced frequency of GC B cells in *Stx11*^−/−^ mice described above (Fig. 2, B and C).

### *Stx11* deficiency impairs T cell**–**dependent B cell responses

Since we detected constitutive expression of STX11 in resting and activated WT CD4 T cells and B cells (Fig. S2 D), we investigated STX11 function in humoral immunity by immunization with either T-independent (TI) or T-dependent (TD) antigens. TI antibody responses are either type 1 (TI-1) or type 2 (TI-2) responses depending on the requirement of the non-receptor kinase BTK for TI-2 responses. Antibody responses to TNP (trinitrophenyl hapten)-LPS (TI-1) are mainly of IgM and after class switch of IgG2 and IgG3 type in similar amounts, which are produced by B cells in extrafollicular sites (Slack et al., 1980). *Stx11*^−/−^ and WT mice mounted comparable TNP-specific IgM and IgG3 responses when immunized with TNP-LPS (Fig. 2 D). B cell responses to TNP-Ficoll (TI-2) are antibody responses due to crosslinking of a critical number of BCRs, primarily on MZ B cells producing mainly IgM and IgG3 (Mongini et al., 1981). No significant differences were observed in IgM or IgG3 titers between *Stx11*^−/−^ and WT mice (Fig. 2 E). Hence, a general B cell defect in *Stx11*-deficient mice could be excluded. To have a comparable TD stimulation, we chose the well-described TNP-KLH (keyhole limpet haemocyanin) system with the Sigma-Aldrich Adjuvant System (Baker et al., 1988). *Stx11*^−/−^ mice exhibited reduced TNP-specific IgG3 antibodies with lower avidity compared with immunized WT mice (Fig. 2 F). Thus, *Stx11-*deficiency specifically impairs B cell responses to TD antigens, suggesting impaired CD4 T cell help. In line with this, *Stx11*-deficient naive B cells of FHL-4 patients differentiated in vitro into plasmablasts and underwent isotype switch after CD40L + IL-21 with or without IL-4 stimulation, mimicking optimal CD4 T cell help (Fig. 2, G and H). In summary, these results excluded a general intrinsic B cell defect in STX11 deficiency.

### Reduced follicular T helper (Tfh) cell frequency during LCMV infection in *Stx11*^*−/−*^ mice

**Figure 3.**
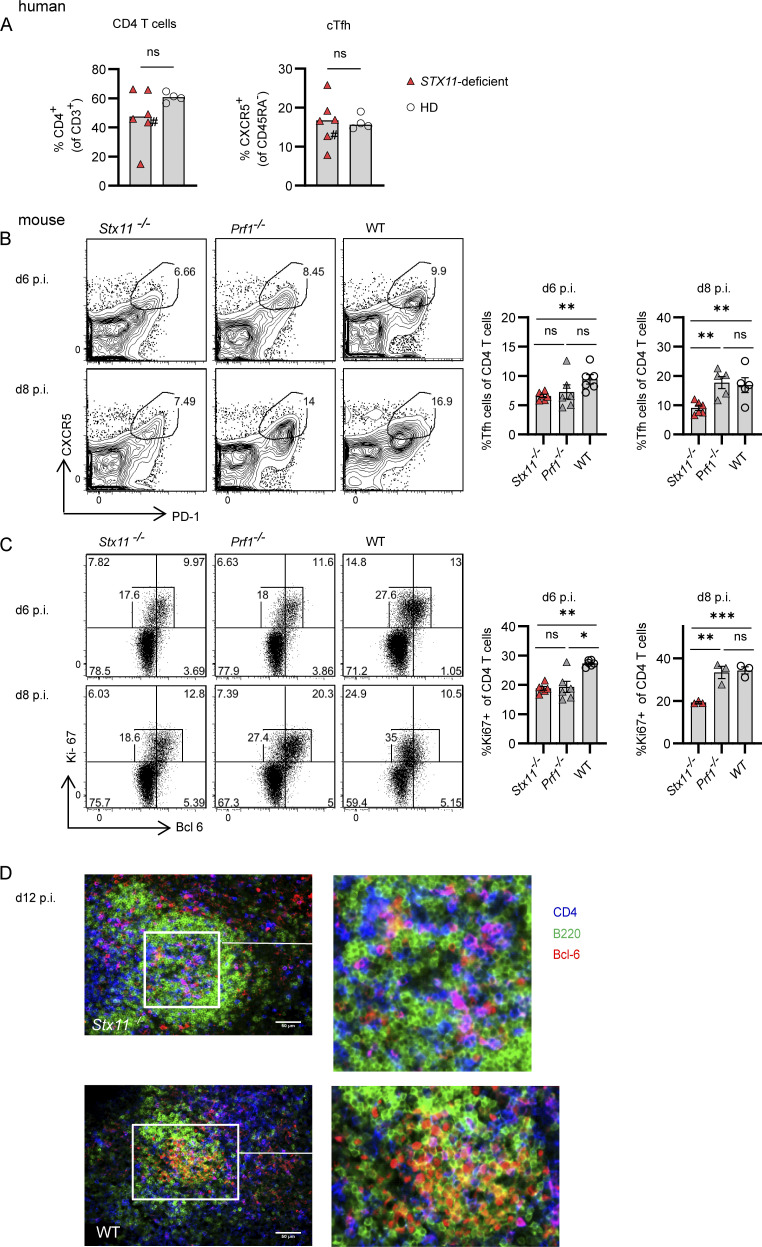
**Reduced Tfh numbers in *Stx11***^***−/−***^ **mice during LCMV infection.** Human: **(A)** Percentages of CD4 T cells and Tfh (CXCR5^+^ CD45RA^−^CD4^+^) cells in frozen PBMCs samples from FHL-4 patients (red triangle) and healthy controls (open circle); #, patient under treatment. Mouse: **(B–D)** *Stx11*^*−/−*^, *Prf1*^*−/−*^, and WT mice infected with 200 PFU LCMV-WE i.v. **(B and C)** (B) Frequency of CXCR5^+^PD-1^+^ Tfh cells *Stx11*^*−/−*^(*n* = 6), *Prf1*^*−/−*^ (*n* = 5–6), and WT (C57BL/6N *n* = 5–6) from *n* = 2 independent experiments and (C) proliferation by Ki-67 expression of Bcl-6^+^ Tfh cells from the spleen of *Stx11*^*−/−*^(*n* = 3 [d8]; *n* = 6 [d6]), *Prf1*^*−/−*^ (*n* = 3 [d8]; *n* = 6 [d6]) and WT (C57BL/6N *n* = 3 [d8]; *n* = 6 [d6]) were analyzed on day 6 (*n* = 2 independent experiments) and d8 (1 experiment) p.i. **(D)** Representative immunofluoresent spleen sections: B220 (green), CD4 (blue), and BCL-6 (red) d12 p.i. with LCMV; WT (C57BL/6N) *n* = 6 of two independent experiments and *Stx11*^*−/−*^ *n* = 3 of two experiments. Scale bar 50 µm. **(A–D)** Mean and ± SEMs of Mann–Whitney *U* test are shown; *P < 0.05, **P < 0.01, *** P < 0.001, ns indicates not significant.

Effective isotype switch, GC reaction, and affinity maturation require the interplay between antigen-presenting cells (APCs), CD4 Tfh cells, and B cells (Crotty, 2015, 2019; Shulman et al., 2013). We observed similar frequencies of CD4 T cells and circulating Tfh cells in FHL-4 patients and healthy controls (Fig. 3 A and gating strategy in Fig. S2 E). To acquire a more dynamic view of CD4 T cell expansion and differentiation, we studied Tfh cells in diseased *Stx11*^*−/−*^ and *Prf1*^*−/−*^ mice persistently infected with LCMV and compared them with non-diseased WT mice, which can clear the virus. No difference in overall CD4 T cell frequencies and numbers was detected between *Stx11*^*−/−*^ and *Prf1*^*−/−*^ mice on d6 and d8 p.i. (Fig. S2 F). Splenic CD4 Tfh cell (CXCR5^+^PD-1^+^) frequencies were similar on d6 p.i. in *Stx11*^*−/−*^ and *Prf1*^*−/−*^ mice, but significantly reduced on d8 in *Stx11*^*−/−*^ mice (Fig. 3 B). Moreover, the frequency of proliferating (Ki-67^+^) CD4 T cells was significantly lower in *Stx11*^*−/−*^ mice on d8 (Fig. 3 C). Immunohistological staining of spleen sections at d12 p.i. showed *Stx11*^*−/−*^ Bcl-6^+^ CD4 T cells (magenta) in the center of B cell follicles (green), suggesting that *Stx11*^*−/−*^ Tfh cells are able to migrate into the B cell follicle, but unable to promote GC formation and maturation of GC B cells (B220^+^Bcl-6^+^, yellow) as seen in WT mice (Fig. 3 D). Thus, reduced interaction of CD4 T cells with B cells might attenuate differentiation, proliferation, and/or maintenance of Tfh cells and therefore lead to impaired GC formation observed in *Stx11*^*−/−*^ mice.

### STX11 expression in B cells is dispensable for antibody responses to LCMV

**Figure 4.**
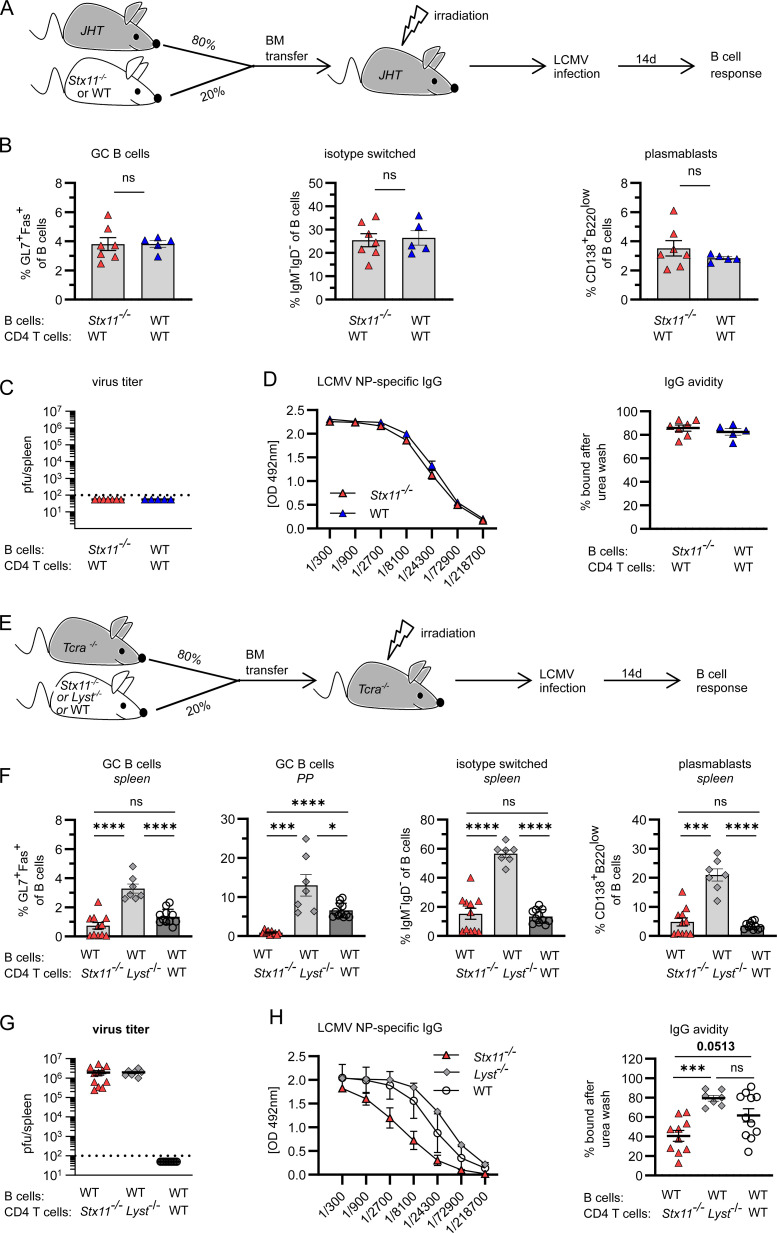
**STX11 expression is not required for functional B cells but for functional CD4 T cells. (A)** Mixed BM chimera: 80% JHT (no B cells) and 20% *Stx11*^*−/−*^ (*n* = 7) or WT CD45.1 C57BL/6J (*n* = 5) BM cells were mixed and injected into irradiated JHT recipients and infected with 200 PFU LCMV-WE 10 wk later. d14 p.i. from *n* = 2 independent experiments. **(B)** GC B cells (Fas^+^GL7^+^), isotype-switched (IgM^−^IgD^−^) B cells, and plasmablasts (CD138^+^) as percentages of CD19^+^B220^+^ B cells in spleens. **(C)** Viral titers in spleen. Dotted line indicates the detection limit. **(D)** LCMV NP-specific IgG levels (left) and IgG avidity in serum (right). **(E)** BM chimera: 80% *Tcra*^*−/−*^ and 20% *Stx11*^*−/−*^ (*n* = 10) or *Lyst*^*−/−*^ BM cells (*n* = 7) or WT CD45.1 C57BL/6J (*n* = 11) in *n* = 2 independent experiments were mixed and injected into irradiated *Tcra*^*−/−*^ recipients and infected with 200 PFU LCMV 8 wk later. d14 p.i. **(F)** Isotype-switched, GC B cells, and plasmablasts as percentages of CD19^+^B220^+^ B cells. **(G)** Viral titers in spleen. **(H)** LCMV NP-specific IgG levels (left) and IgG avidity in serum (right). For avidity measurements, antigen-captured serum IgG was treated with PBS or 8 M urea. **(A–H)** Mean and ± SEMs of Mann–Whitney *U* test are shown; depending on normal distribution *P < 0.05, *** P < 0.001, **** < 0.0001, ns indicates not significant.

To further investigate the requirement of STX11 expression for B cell differentiation, we generated mixed BM chimeras. JHT mice, constitutively lacking B cells, were irradiated and reconstituted with a mixture of 80% JHT BM and 20% *Stx11*-deficient BM generating *Stx11*-deficient B cells and mostly *Stx11*-positive CD4 T cells (*Stx11*-deficient B cell chimeras). As a control, we established BM chimeras with 80% JHT and 20% WT BM generating *Stx11*-positive B and CD4 T cells (*Stx11*-positive B cell chimeras) (Fig. 4 A). No differences in frequencies of GC B cells, plasmablasts, and isotype-switched B cells were detected in the two experimental groups upon LCMV infection (Fig. 4 B). Both chimera groups controlled LCMV (Fig. 4 C) and mounted a strong NP-specific IgG response with comparable IgG avidity (Fig. 4 D). The experiment supports our findings that *Stx11* deficiency in B cells is inconsequential to their function.

To assess a T cell–intrinsic function of STX11, *Tcra*-deficient mice constitutively lacking T cells were irradiated and reconstituted with a mixture of 80% *Tcra*^−/−^ and 20% *Stx11*^−/−^ BM, generating chimeras with *Stx11*-deficient T cells and mostly *Stx11*-positive B cells (*Stx11*-deficient T cell chimeras). Since *Stx11*^−/−^ CD8 T cells have a degranulation defect, we compared them with T cells from *Lyst-*deficient mice that also show a degranulation defect, impaired viral control, and similar HLH development. Mice were reconstituted with 80% *Tcra*-deficient BM and 20% BM from *Lyst-*deficient mice, generating *Stx11*-positive CD4 T and B cells (*Stx11*-positive T cell chimeras) in the presence of cytotoxicity-deficient (*Lyst*-deficient) CD8 T cells (Fig. 4 E). *Stx11*-deficient T cell chimeras showed significantly impaired B cell responses with reduced frequencies of GC B cells, isotype-switched B cells, and plasmablasts in comparison with *Stx11*-positive (*Lyst*-deficient) T cell chimeras (Fig. 4 F) under hyperinflammatory conditions. As expected, in both experimental groups, the virus persisted, leading to high and prolonged antigen exposure (Fig. 4 G). *Stx11*-deficient T cell chimeras mounted a weaker NP-specific IgG antibody response with lower avidity (Fig. 4 H). Additionally, we included *Tcra*-deficient mice reconstituted with 80% *Tcra*-deficient BM and 20% BM from WT as controls. Importantly, these chimeras did not develop hyperinflammation and cleared the virus (Fig. 4 G), which impacts the local cytokine milieu and antigen availability. However, B cell differentiation significantly improved in Peyer’s patches, but not in spleens of these WT chimeras. In summary, the BM chimeras demonstrate an intrinsic role of the SNARE protein STX11 in CD4 T cell help for B cells.

### STX11 expression is required for CD4 T cell help

**Figure 5.**
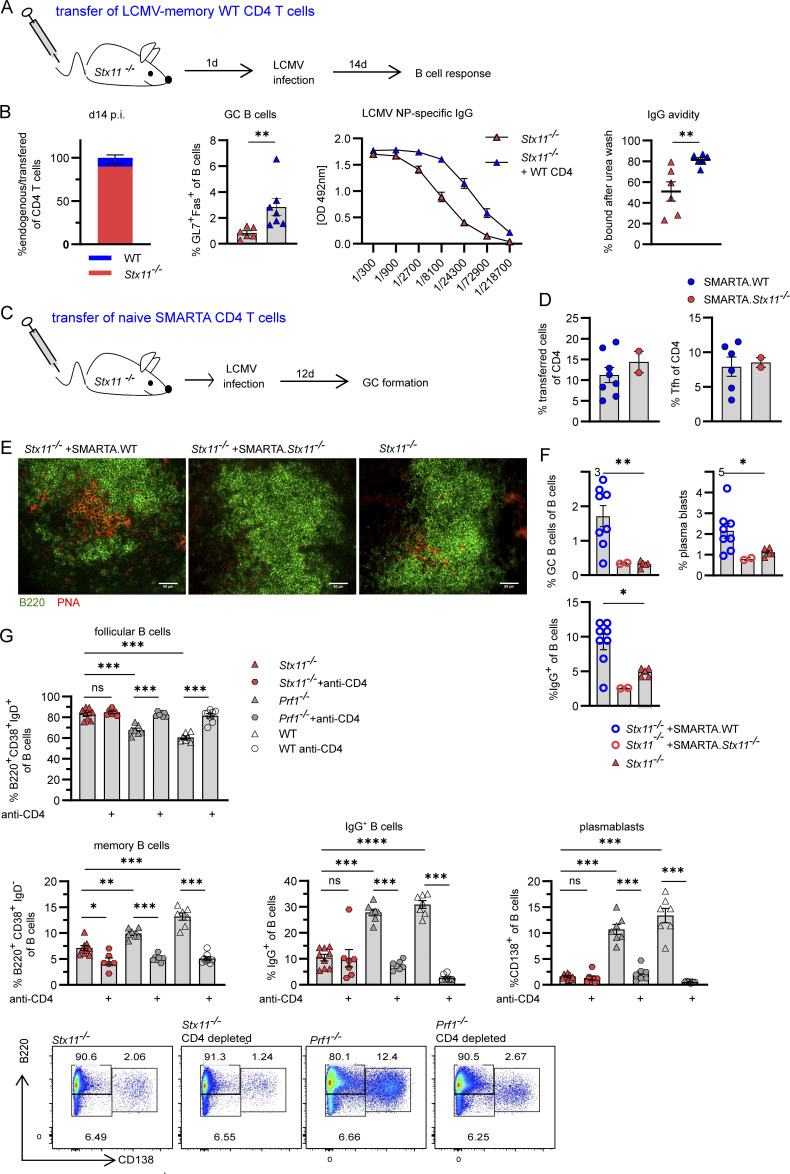
**Secondary B cell defect due to limited CD4 T cell help in *Stx11* deficiency. (A)** Experimental setup for adoptive transfer (AdTf) of WT.CD45.1 (LCMV-memory CD4 T cells) in *Stx11*^*−/−*^ mice. On d3 and d1 prior to AdTf, *Stx11*^*−/−*^ mice were depleted of CD8 T cells by antibody injection. *Stx11*^*−/−*^ mice with AdTf (+ WT.CD45.1 [C57BL/6J] CD4, *n* = 7) or without (*n* = 6) were infected with 200 PFU LCMV i.v. (*n* = 2 independent experiments). **(B)** Far left: The proportion of transferred CD4 T cells in spleen d14 p.i. Middle left: Percentages of GC B cells (GL7^+^Fas^+^ of CD19^+^B220^+^). Middle right: LCMV NP-specific IgG in serum quantified by ELISA. Far right: IgG avidity: antigen-captured serum IgG was washed with PBS or 8 M urea. The relative avidity of NP-specific IgG shown as AUC with and without urea treatment (% AUC = AUC+urea/AUC+PBS*100). **(C)** 10^6^ purified naive SMARTA CD4 T cells were transferred into CD8 T cell–depleted *Stx11*^*−/−*^ mice and infected with LCMV and analyzed at d12 p.i. **(D)** Percentage of transferred SMARTA.WT (*n* = 8, blue) or SMARTA.*Stx11*^*−/−*^ (*n* = 2, red) CD4 T cells and percentage of SMARTA.WT or SMARTA.*Stx11*^*−/−*^ Tfh (CXCR5^+^PD1^+^) cells of the CD4 T cell compartment. **(E)** Representative spleen section showing GC formation after SMARTA.WT transfer: B220 (green), PNA (red) (*Stx11*^*−/−*^ *n* = 4, *Stx11*^*−/−*^ +SMARTA.WT *n* = 4, *Stx11*^*−/−*^ +SMARTA*.Stx11*^*−/−*^ *n* = 2 of two independent experiments). Scale bar 50 µm. **(F)** Percentages of GC (Fas^+^GL7^+^), plamablasts (CD138^+^), and IgG^+^ B cells of *Stx11*^*−/−*^ with SMARTA.WT (blue open circles), SMARTA.*Stx11*^*−/−*^ (red open circles), or without transfer (red triangle). **(G)** B cell differentiation (follicular B cells, memory B cells, IgG^+^ B cells, and plasmablasts) ± CD4 T cell depletion (day −3 and −1) in *Stx11*^*−/−*^ (*n* = 9 and *n* = 7 CD4 depleted), *Prf1*^*−/−*^ (*n* = 7 and *n* = 7 CD4 depleted), and WT (C57BL/6N) mice (*n* = 8 and *n* = 7 CD4 depleted) mice after 200 PFU LCMV infection on d12–17 p.i. from *n* = 2 independent experiments for each group. Bottom: Representative FACS plots showing plasmablast (CD138^+^) differentiation d12 p.i. **(A–G)** Mean and ± SEMs of Mann–Whitney *U* test are shown; *P < 0.05, **P < 0.01, *** P < 0.001, **** < 0.0001, ns indicates not significant.

Based on our findings from the mixed BM chimeras, we hypothesize that transfer of a low number of WT-memory CD4 T cells into *Stx11*^−/−^ mice may rescue the impaired B cell response. CD4 T cells from LCMV-immune WT mice were transferred into *Stx11*^−/−^ mice. CD8 T cells were depleted prior to transfer to limit hyperinflammation (Fig. 5 A). Reconstitution of ∼15% WT CD4 T cells in the splenic CD4 T compartment (Fig. 5 B) resulted in significantly higher frequency of GC B cells and higher NP-specific IgG-levels with increased high-avidity antibodies (Fig. 5 B) at day 14 p.i., showing that functional CD4 T cells can rescue the secondary B cell defect in *Stx11*^−/−^ mice. As an additional approach, we transferred naive TCR-transgenic LCMV-GP_61-80_-specific SMARTA.WT or SMARTA.*Stx11*^*−/−*^ CD4 T cells into CD8 T cell–depleted *Stx11*^−/−^ mice (Fig. 5 C) and analyzed GC formation by immunohistology and B cell differentiation. GC formation was successful in spleens of mice with SMARTA.WT CD4 T cell transfer but not in spleens of mice with SMARTA.*Stx11*^*−/−*^ CD4 T cell transfer or no transfer control (Fig. 5 E). In correlation with the frequency of transferred SMARTA.WT CD4 T cells (Fig. 5 D and Fig. S2 G), the percentage of GC B cells, plasmablasts, and IgG^+^ B cells increased significantly compared with *Stx11*^*−/−*^ mice without transfer or SMARTA.*Stx11*^*−/−*^ CD4 T cell transfer (Fig. 5 F).

To assess if STX11-deficient CD4 T cells retained residual functions relevant to B cell help, CD4 T cells were depleted in *Stx11*^−/−^, *Prf1*^*−/−*^, and WT mice. B cell responses were analyzed after LCMV infection. Remarkably, *Stx11-*deficient mice with and without depletion of CD4 T cells exhibited comparable frequencies of follicular (B220^high^IgD^high^CD38^+^), isotype-switched IgG^+^ B cells and plasmablasts (CD138^+^) (Fig. 5 G). Untreated *Prf1*^*−/−*^ and WT mice mounted a strong B cell response with B cell activation, differentiation, and isotype switching (Fig. 5 G). In contrast, CD4 T cell depletion in *Prf1*^*−/−*^ and WT mice resulted in a B cell phenotype comparable with the phenotype seen in *Stx11*-deficient mice. In summary, the level of help provided by *Stx11*-deficient T cells is functionally equivalent to a complete loss of CD4 T cell function.

### *Stx11*^−/−^ CD4 T cells are impaired in promoting differentiation of WT B cells in vitro

**Figure 6.**
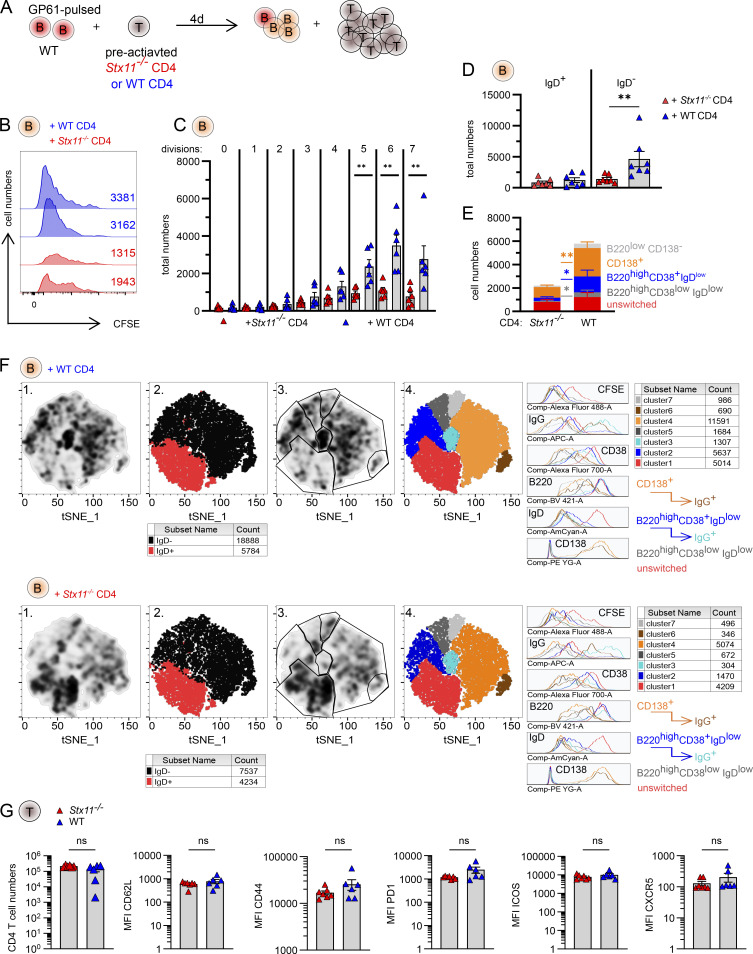
***Stx11***^***−/−***^ **CD4 T cells are limited to help WT B cells in vitro*.* (A)** Experimental setup of in vitro T/B cell interaction assay with naive, LCMV-GP_61-80_-pulsed WT (C57BL/6N) B cells incubated with d6 pre-activated SMARTA.WT or SMARTA.*Stx11*^*−/−*^ CD4 T cells. **(B)** Two representative histograms of proliferating (d4 CFSE dilution) WT B cells in coculture with either SMARTA.WT (blue) or SMARTA.*Stx11*^*−/−*^ (red) CD4 T cells. **(C)** Total B cell numbers for different cell divisions (0 = no proliferation; 7 = 7 cell divisions). SMARTA.WT *n* = 6 and SMARTA.*Stx11*^*−/−*^ *n* = 6 from *n* = 4 independent experiments. **(D)** Absolute numbers of unswitched (IgD^+^) versus isotype-switched (IgD^−^) B cells on d4 of co-culture of WT B cells with SMARTA.*Stx11*^*−/−*^ *n* = 7 or SMARTA.WT *n* = 7 from *n* = 5 independent experiments. **(E)** Absolute numbers of unswitched B cells (B220^high^IgD^+^CD38^+^CD138^−^, red), GC-like B cells (B220^high^IgD^low^CD38^low^, dark gray), memory-like B cells (B220^high^IgD^low^CD38^+^, blue), and plasmablast-like (CD138^+^) B cells after co-culture with either SMARTA.*Stx11*^*−/−*^ (*n* = 7) or SMARTA.WT (*n* = 7) CD4 T cells for 4 days (*n* = 5 independent experiments). **(F)** Pooled WT B cells (lymphocytes → single cells → live cells → CD4^−^ B220^+^) were cocultured with either SMARTA.WT (top, *n* = 6) or SMARTA.*Stx11*^*−/−*^ CD4 T cells (bottom, *n* = 6) in four independent experiments and analyzed with t-SNE. (1) Density pattern for B cells. (2) Distribution of unswitched (IgD^+^, red) versus isotype-switched (IgD^−^, black) B cells. (3) Gating of different clusters according to their density and marker expression patterns. (4) Marker expression of clusters identified: unswitched (IgD^+^, red), GC-like (B220^high^IgD^low^CD38^low^, gray), memory-like (B220^high^IgD^low^CD38^+^, blue), IgG^+^ memory-like (B220^high^IgD^low^CD38^+^, cyan), plasmablast-like (CD138^+^, orange), and IgG^+^ plasmablast-like (CD138^+^, brown) B cells and cells with low expression of all markers (light gray). **(G)** Total SMARTA.*Stx11*^*−/−*^ or SMARTA.WT CD4 T cells at d4 of co-culture with WT B cells. Median fluorescence intensity (MFI) of follicular CD4 T cell markers expressed by SMARTA.*Stx11*^*−/−*^ (*n* = 6) or SMARTA.WT (*n* = 6) after 4 days of co-culture with GP_61-80_-pulsed WT B cells from *n* = 4 independent experiments. **(A–G)** Mean and ± SEMs of Mann–Whitney *U* test are shown; **P < 0.01, ns indicates not significant.

**Figure S3.**
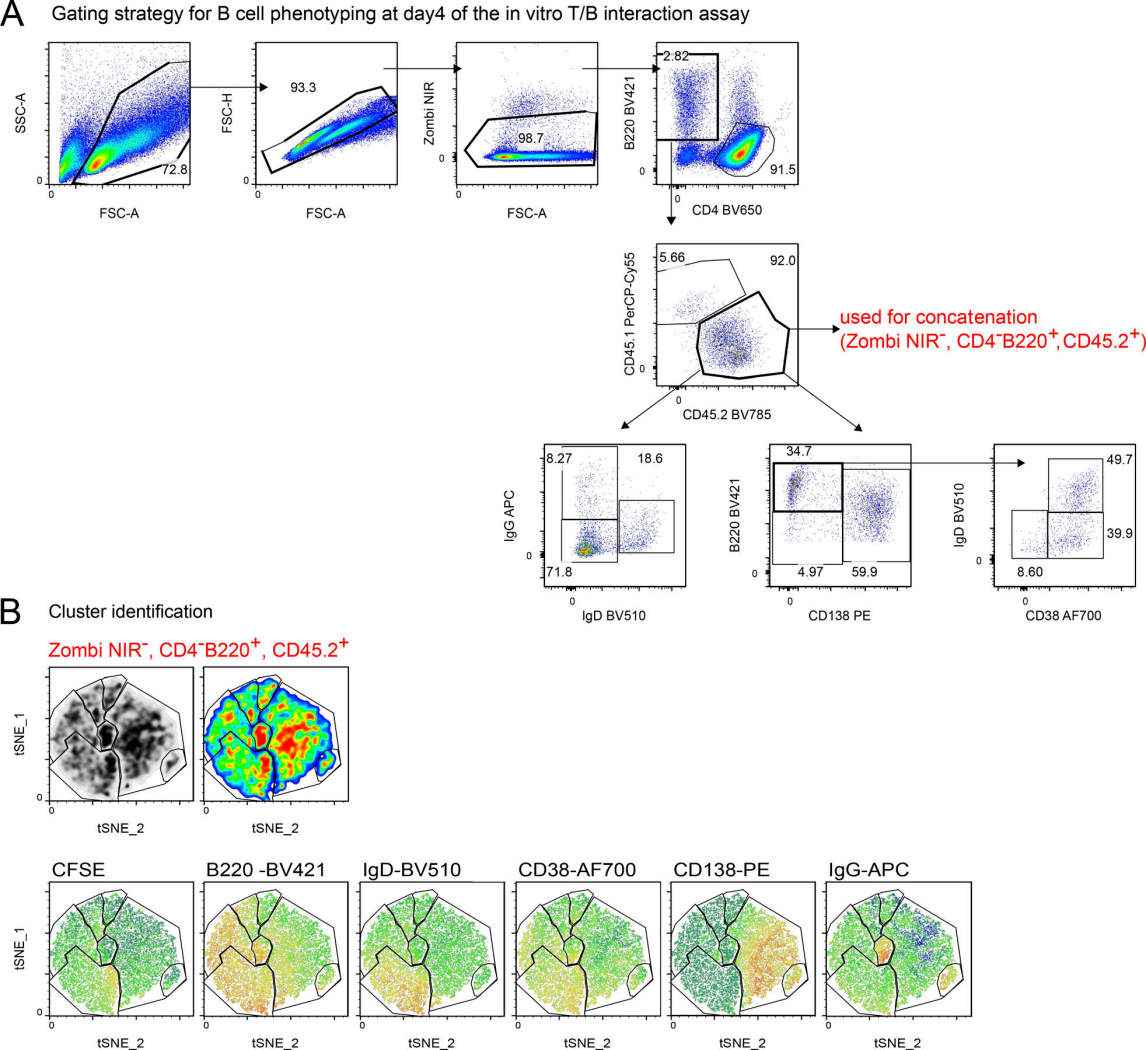
**Cluster identification for t-SNE analysis. (A)** Pooled data from B cells co-cultured with SMARTA.WT and SMARTA.*Stx11*^*−/−*^ were analyzed with t-SNE: perplexity: 20, itinerary: 2,000. Pre gates for B cells were defined (lymphocytes → single cells → live cells → CD4^−^ B220^+^) to exclude dead cells and CD4 T cells. **(B)** Clusters were identified and gated by correlation of density plot, pseudocolor plot, and marker distribution.

To study the differentiation of WT B cells in the presence of WT or *Stx11*^−/−^ CD4 T cells under non-inflammatory conditions, we established an antigen-specific in vitro T/B cell interaction assay. We used TCR-transgenic LCMV-GP_61-80_-specific SMARTA.WT or SMARTA.*Stx11*^−/−^ CD4 T cells, which were preactivated for 6 days with plate-bound anti-CD3/soluble anti-CD28 antibodies before co-culture with LCMV-GP_61-80_–pulsed naive WT B cells (Fig. 6 A). B cell proliferation (CFSE dilution) and isotype switching were analyzed at d4 in this in vitro model of cognate T-B interaction (Fig. 6, B–E). WT B cells receiving help from *Stx11*^−/−^ CD4 T cells were activated and entered the cell cycle; however, their numbers were significantly reduced compared to B cells with help from WT CD4 T cells (Fig. 6, B and C). The total number of isotype-switched B cells (IgD^−^) was higher when B cells had help from WT CD4 T cells (Fig. 6 D). Analysis of B cell subpopulations showed an increased proportion of activated unswitched (B220^high^CD38^+^IgD^+^) and reduced numbers of isotype-switched B cells, divided into GC-like (B220^high^CD38^low^IgD^low^), memory-like (B220^high^CD38^+^IgD^low^), and plasmablast-like (CD138^+^) B cells after coculture with *Stx11*^−/−^ CD4 T cells (Fig. 6 E). Unsupervised t-distributed stochastic neighbor embedding (t-SNE) analysis of pooled data from all experiments confirmed a different density distribution pattern of B cell subsets stimulated with WT versus *Stx11*^*−/−*^ CD4 T cells (Fig. 6 F, column 1, and gating strategy in Fig. S3 A). Clusters were identified by a combination of density and marker distribution of all B cells independently of help from WT or *Stx11*^−/−^ CD4 T cells (Fig. S3 B). Clusters correlating with the highest density of B cells with help from WT CD4 T cells were found in the isotype-switched, IgD^−^ cluster (black), whereas higher density of B cells with help from *Stx11*^*−/−*^ CD4 T cells were found in the unswitched, IgD^+^ cluster (red) (Fig. 6 F, column 2). Gating of different subclusters identified unswitched (red), GC-like (gray), memory-like (blue), and plasmablast-like (orange) B cell subpopulations. IgG^interm.^ B cells (cyan) were found in the memory-like cluster and IgG^high^ B cells (brown) in the plasmablast-like (CD138^+^) compartment (Fig. 6 F, columns 3 and 4). Interestingly, comparison of WT versus *Stx11*^*−/−*^ CD4 T cell phenotypes on d4 of T/B cell interaction did not show any significant differences in CD4 T cell proliferation with similar numbers and surface expression levels of CD62L, CD44, PD1, ICOS, or CXCR5 (Fig. 6 G). In summary, the newly established in vitro T/B cell interaction assay under standardized, non-inflammatory conditions demonstrated that *Stx11-*deficient CD4 T cells are unable to support the expansion, differentiation, or maintenance of WT B cells. Nevertheless, the WT B cells receiving help from *Stx11-*deficient CD4 T cells were activated but did not undergo isotype switching and overall B cell numbers were lower.

### Impaired CD107a and CD40L mobilization in CD4 *Stx11*^*−/−*^ T cells

**Figure 7.**
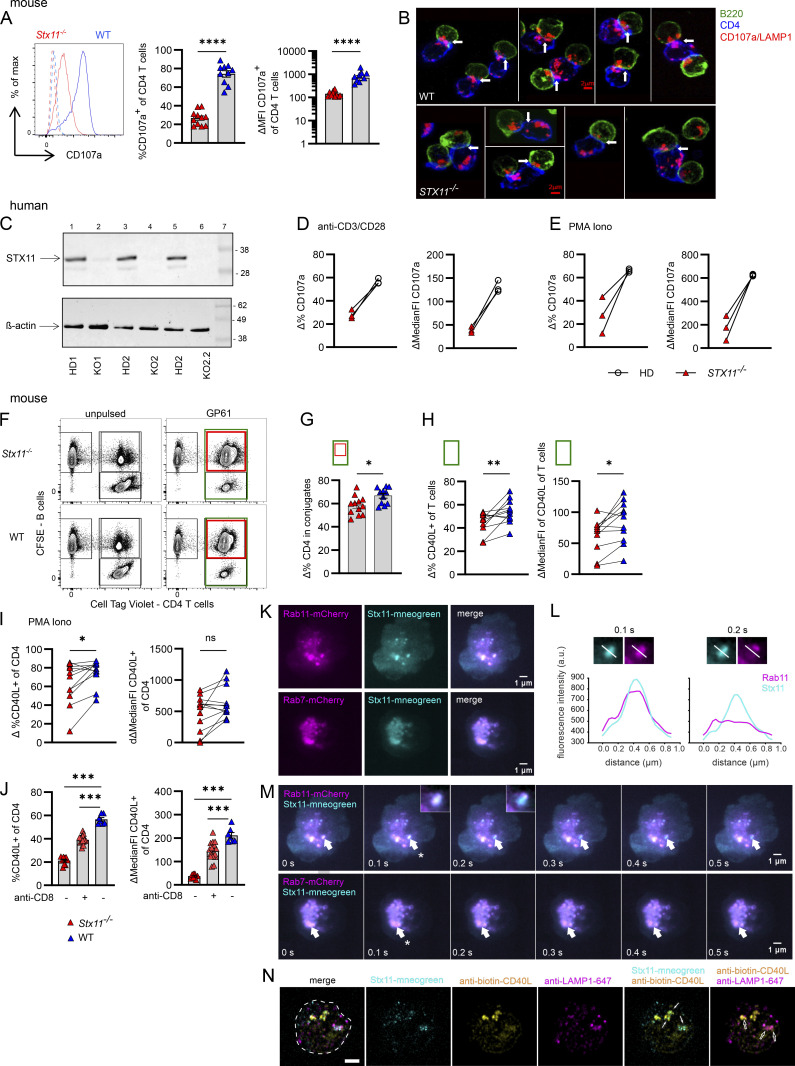
**Impaired mobilization of CD107a and CD40L in *Stx11***^***−/−***^ **CD4 T cells.** Mouse: **(A)** d6 pre-activated SMARTA.*Stx11*^*−/−*^ (red, *n* = 11) or SMARTA.WT (blue, *n* = 10) (*n* = 8 independent experiments) were re-stimulated with plate-bound anti-CD3 (10 µg/ml) for 2 h in the presence of anti-CD107a antibodies. Surface CD107a was measured by flow cytometry. Representative overlay of histograms (left), delta of stimulated minus medium-only of percentage (middle), and median fluorescence intensity (MFI) (right). **(B)** Mobilization of CD107a^+^ vesicles (red) of CD4 T cells (blue) with LCMV-GP_61-80_-pulsed WT B cells (green) after 35 min incubation (WT *n* = 2, *Stx11*^*−/−*^ *n* = 2, one experiment). Scale bar 2 µm; arrows indicate immunological synapses. Human: **(C)** Western blot for STX11 expression in CD4 T cells of two healthy donors (HD) and after CRISPRCas9 KO of STX11 using four RNP guides (KO1+KO2) or six RNP guides (KO2.2) at d10 of restimulation with anti-CD3/CD28/CD2 beads and rhIL-12. Molecular mass is shown in kilodaltons. **(D)** Re-stimulation of d10 cultured human CD4 T cells for 3.5 h with anti-CD3 and soluble anti-CD28 (both 2 µg/ml) (representative experiment of *n* = 2). **(E)** Restimulation of d10 cultured human CD4 T cells for 3.5 h with PMA/Iono (representative experiment of n = 2). Mouse: **(F)** Representative dot plots of conjugation assay of CFSE-labeled, LPS-activated, GP_61-80_-pulsed or unpulsed WT B cells with d7 pre-cultured SMARTA.CD4 T cells. **(G)** Percentage of CD4.SMARTA.*Stx11*^*−/−*^ (red, *n* = 12) or CD4.SMARTA.WT (blue, *n* = 11) in conjugates with B cells (from F [red/green] ×100%) after 30 min incubation (*n* = 8 independent experiments). **(H)** CD40L mobilization: CD40L^+^ T cells (as % of total T cells) shown as Δ of stimulated with GP_61-80_-pulsed minus unpulsed WT B cells (green rectangle shown in F) (left) or CD40L MFI on CD4 T cells (green rectangle shown in F) (right). SMARTA.*Stx11*^*−/−*^ (red, *n* = 12) or SMARTA.WT (blue, *n* = 11) from *n* = 8 independent experiments. **(I)** CD40L mobilization: Δ of PMA/Iono stimulated minus medium-only of d6–7 pre-cultured CD4 T cells. Incubation time 30 min, showing percentage of CD40L^+^ CD4 T cells (left) and MFI of CD40L (right). SMARTA.*Stx11*^*−/−*^ (red, *n* = 12) or SMARTA.WT (blue, *n* = 11) from *n* = 8 independent experiments. **(J)** *Stx11*^*−/−*^ (*n* = 8), CD8 T cell–depleted (anti-CD8 +) *Stx11*^*−/−*^ (*n* = 16), and WT (C57BL/6N) mice (*n* = 7) were infected with 200 PFU LCMV. Cells were isolated d12 p.i. and re-stimulated with PMA/Iono. Δ = PMA/Iono minus medium-only control. *n* = 2 independent experiments. **(K)** Snapshots from live TIRF imaging of SMARTA.WT CD4 T cells co-transfected with *Stx11*-mNeonGreen and either *Rab11-*mCherry or *Rab7*-mCherry constructs were captured. Cells were positioned on anti-CD3–coated coverslips to observe vesicle polarization and fusion at the synapse. Scale bar 1 µm. **(L)** Fusion profile analysis at 0.1 and 0.2 s of STX11 and RAB11. Scale bar 1 µm. **(M)** The video timestamps (Video 1 and Video 2) of the captured frames are shown on the bottom left of each image. Fusion events are indicated by white arrows. Timestamp when a fusion event happens is marked by an asterisk. Scale bar 1 µm. **(N)** SIM images of a representative WT CD4 T cell transfected with *Stx11*-mNeonGreen construct. Cells were incubated on anti-CD3–coated coverslips for 30 min, allowing the formation of a synapse and vesicle release. Fixed cells were stained with biotinylated anti-CD40L and anti-LAMP-1 antibodies to analyze co-localization using SIM. Images were acquired at the synapse area above the coverslips. All images were subsequently analyzed and presented after post-processing. The footprint of the cell is marked by white stipple line. Solid arrows point to the colocalization of STX11 and CD40L, whereas the opened arrows point to the colocalization of LAMP1 and CD40L. Scale bar 2 µm. **(A–J)** Mean and ± SEMs of Mann–Whitney *U* test are shown; *P < 0.05, **P < 0.01, *** P < 0.001, **** < 0.0001, ns indicates not significant. Source data are available for this figure: SourceData F7.

Since the surface presentation of various Tfh cell markers on *Stx11*^*−/−*^ versus WT CD4 T cells were comparable after T/B cell interaction (Fig. 6 G), we assessed vesicle transport in CD4 T cells. First, we investigated if CD4 T cells exocytose CD107a. Indeed, WT CD4 T cells showed exocytosis of CD107a^+^ lysosomal vesicles after re-stimulation with anti-CD3, reminiscent of CD8 T cell degranulation, whereas CD107a mobilization was strongly impaired in *Stx11*^*−/−*^ CD4 T cells (Fig. 7 A). We further investigated whether the trafficking of CD107a^+^ vesicles in CD4 T cells upon B cell contact is affected in the absence of STX11. Immunofluorescence staining of T/B cell interaction and localization of CD107a^+^ vesicles (red) in CD4 T cells (blue) demonstrated impaired polarization to the immunological synapse (IS, arrow) in *Stx11*^*−/−*^ CD4 T cells compared with WT CD4 T cells. As the IS recruits numerous signaling molecules triggering vesicle secretion in both T cells and B cells, successful T/B cell interaction can be judged by CD107a^+^ vesicle polarization to the synapse in B cells (green) (Fig. 7 B) (Obino et al., 2017). To verify that impaired CD107a mobilization is not limited to mouse *Stx11*^*−/−*^ CD4 T cells, we utilized a CRISPR-Cas9 approach to knock out (KO) STX11 in two healthy human donors (Fig. 7 C) and restimulated with either anti-CD3/CD28 (Fig. 7 D) or PMA/Ionomycine (Iono) (Fig. 7 E). Flow cytometry results showed a significantly reduced CD107a exocytosis in STX11-KO human CD4 T cells, underscoring the crucial role of STX11 in both lysosome transportation and secretion.

Next, we examined if STX11 affects T/B cell conjugate formation and stability. We precultured *Stx11*-deficient or WT CD4 T cells (d6) and mixed them with LPS-activated WT B cells, pulsed with GP_61-80_ peptide. After 30 min interaction time, fewer *Stx11*^*−/−*^ CD4 T cells were found in conjugation with B cells when compared with WT CD4 T cells (Fig. 7, F and G). Since CD40L is critical for B cell survival and differentiation and is described to be stored in lysosomal compartments in CD4 T cells (Koguchi et al., 2007; Jolly and Sattentau, 2007; Casamayor-Palleja et al., 1995), CD40L expression was investigated after B cell interaction. The overall percentage of CD40L^+^ *Stx11*^*−/−*^ CD4 T cells was reduced and accompanied by a lower mean fluorescence intensity of CD40L on these cells compared with WT CD4 T cells (Fig. 7 H). Percentages of CD40L^+^ *Stx11*^*−/−*^ CD4 T cells were also lower when restimulated with PMA/Iono, in comparison with CD40L^+^ WT CD4 T cells (Fig. 7 I). To exclude that the observed difference in CD40L mobilization was only seen in SMARTA.CD4 T cell cultures, we investigated CD40L mobilization of polyclonal CD4 T cells after in vivo activation with LCMV (d12 p.i.) with or without CD8 T cell depletion (non- or hyperinflammatory conditions, respectively) in *Stx11*^*−/−*^ mice in comparison with WT. The *Stx11*^*−/−*^ or WT CD4 T cells were restimulated ex vivo with PMA/Iono (Fig. 7 J). Both *Stx11*^*−/−*^ CD4 T cell groups showed significantly lower percentages of CD40L^+^ CD4 T cells and decreased median fluorescence intensity in comparison with WT CD4 T cells (Fig. 7 J). CD40L can either be presented on the cell surface or transported to the synapse through secretory lysosomes or via release through extracellular vesicles (Saliba et al., 2019; Cespedes et al., 2022; Koguchi et al., 2007). To better understand the intracellular transport mechanisms leading to STX11 accumulation at the immunological synapse, we visualized the transport of STX11 to the synapse by cotransfecting WT CD4 T cells with *Stx11*-mNeongreen and either *Rab11*-mCherry or *Rab7*-mCherry constructs. RAB11 labels recycling endosomes and RAB7 is a marker for multivesicular bodies (MVBs), known to carry exosomes. Live-cell total internal reflection fluorescence (TIRF) live imaging demonstrated that STX11 is transported by recycling endosomes (RAB11^+^) and late endosomes (RAB7^+^) to the immunological synapse (Fig. 7 K), fusing with the synaptic membrane (Fig. 7 M, asterisk, and Videos 1 and 2). The fusion profile showed the collapse of the STX11^+^RAB11^+^ vesicle from 0.1 to 0.2 s in the recording and revealed a loss of RAB11 fluorescence signal (magenta), but sustained STX11 fluorescence signal (cyan) (Fig. 7 L), suggesting that STX11 may create hotspots to support SNARE-mediated fusion at the plasma membrane. This finding aligns with the observation in human cytotoxic CD8 T cells, where STX11 is partially carried by RAB11^+^ recycling endosomes and fuses at the synapse to mediate T cell effector function by forming secretory hotspots for lytic granule fusion (Halimani et al., 2014). To further investigate the role of STX11 in CD40L release at the synapse, we utilized super-resolution structured illumination microscopy (SIM) to study the colocalization of CD40L, CD107a/LAMP1, and STX11. We observed partial colocalization of accumulated STX11, CD40L, and LAMP1 (Fig. 7 N), suggesting a potential prerequisite role of STX11 and LAMP1 in CD40L secretion at the synapse. These results demonstrate that STX11 impacts overall membrane trafficking and exocytosis in CD4 T cells, affecting at least one key molecule, CD40L, at the synapse, and thereby influencing T/B cell interaction.

**Video 1.** **Localization at the IS of STX11 and RAB11.** 100 frames per second (fps).

**Video 2.** **Localization of STX11 and RAB7 in WT CD4 T cells.** 100 fps.

### Reduced IL-2 and IL-10 release by *Stx11*^*−/−*^ CD4 T cells further impairs T/B interaction

**Figure S4.**
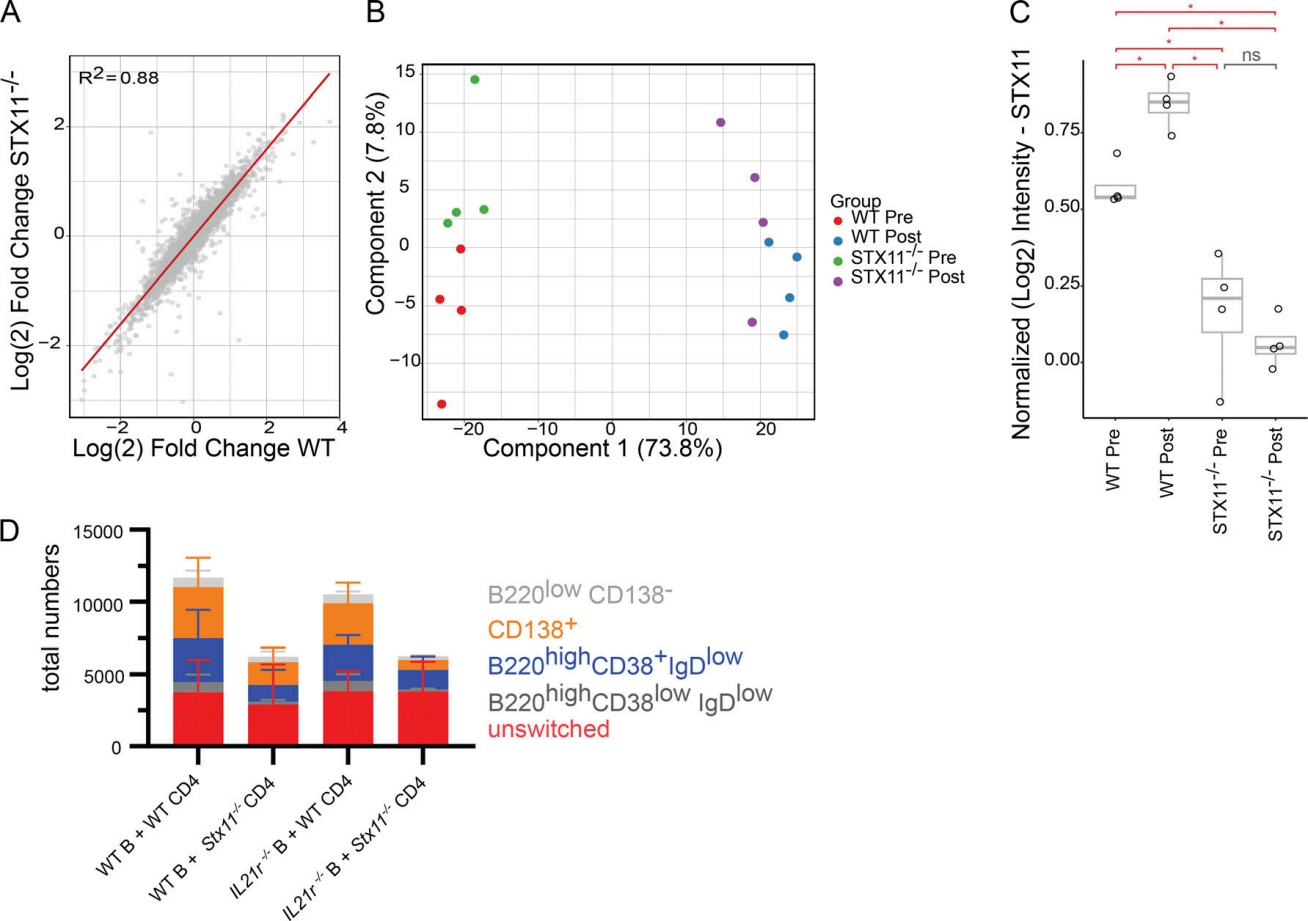
**Protein analysis and IL-21 exclusion. (A)** Comparison of relative protein abundance changes (Log_2_ fold change) in SMARTA.*Stx11*^*−/−*^ CD4 T cells versus SMARTA.WT CD4 T cells after 3 days of in vitro T/B cell interaction. Proteome alterations induced by T/B cell interaction are remarkably consistent between cell lines (trend line = red line, R2 = 0.88). **(B)** PCA demonstrates high reproducibility of the assay with samples grouping according to genotype and stimulation. **(C)** Comparison of normalized STX11 levels between samples (*n* = 4 all groups). Red bars indicate significance at P < 0.05 (*) (one-way ANOVA and Tukey post-hoc mean comparison). Gray bar indicates not significant (ns) at P < 0.05 level. **(D)** Differentiation of WT or IL21r-deficient (*IL21r*^*−/−*^) B cells at d4 of T-B cell interaction assay with either SMARTA.WT CD4 T cells (*n* = 5) or SMARTA.*Stx11*^*−/−*^ CD4 T cells (*n* = 4) (*n* = 3 independent experiments).

**Figure 8.**
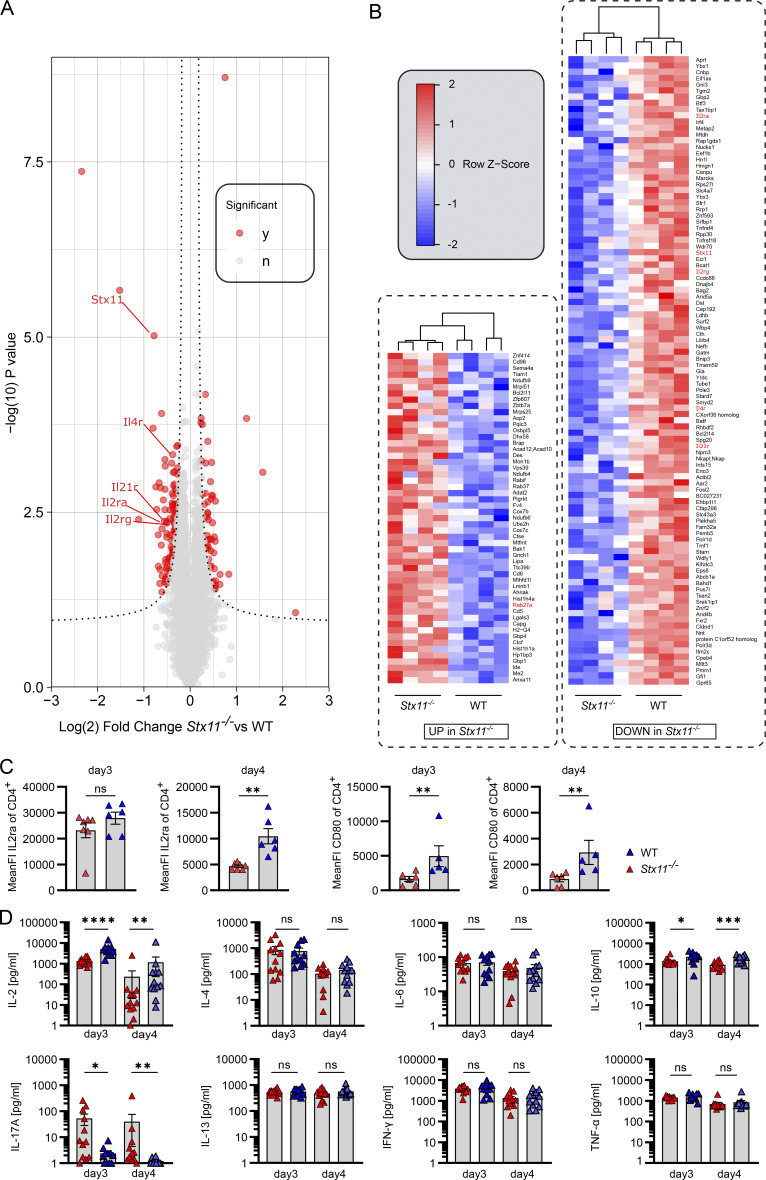
**Reduced IL-2 and IL-10 release and IL2ra expression by *Stx11***^***−/−***^ **CD4 T cells. (A)** Volcano plot of relative protein abundance from mass spectrometric analysis of total protein isolates from sorted SMARTA.*Stx11*^*−/−*^ (*n* = 4) or SMARTA.WT (*n* = 4) CD4 T cells on day 3 of in vitro T/B interaction (*n* = 3 independent experiments). The dashed line represents statistical significance at an estimated FDR < 0.05 (two-tailed *t* test plus permutation-based FDR correction with S0 = 0.1). Significantly altered proteins shown in red. **(B)** Heat map showing proteins being up- or downregulated in SMARTA.*Stx11*^*−/−*^ in comparison to SMARTA.WT after B cell interaction. **(C)** Mean fluorescence intensity of surface IL2ra and CD80 on CD4 T cells on d3 and d4 of T/B interaction. CD4.SMARTA.*Stx11*^*−/−*^ (*n* = 6–7) or CD4.SMARTA.WT (*n* = 5–6) (IL2ra *n* = 4 and CD80 *n* = 3 independent experiments). **(D)** Multiplex analysis of cytokines in the supernatant of d3 and d4 of T/B interaction co-cultures. CD4.SMARTA.*Stx11*^*−/−*^ (*n* = 12) or CD4.SMARTA.WT (*n* = 11–12) of *n* = 8 independent experiments. **(B–D)** Mean and ± SEMs of Mann–Whitney *U* test are shown; *P < 0.05, **P < 0.01, *** P < 0.001, **** < 0.0001, ns indicates not significant.

To elucidate differences in protein expression profiles between WT and *Stx11*^*−/−*^ CD4 T cells, we conducted MS analysis on the T cell proteome collected at d0 (before B cell interaction) and d3 (after B cell interaction) during an in vitro T/B cell interaction assay. The setup prevents variations in in vivo activation, like antigen load and inflammatory conditions, by using anti-CD3/CD28 for preactivation and the same peptide-loaded WT B cells. Overall, the proteome alterations induced by B cell interaction were remarkably consistent between SMARTA.WT and SMARTA.*Stx11*^*−/−*^ CD4 T cells (Fig. S4 A), indicating the absence of a generalized functional CD4 T cell defect in *Stx11*^*−/−*^ as compared with WT cells. In line with this, many transcription factors, important for CD4 helper T (Th)1, Th2, and Tfh cells were comparably expressed, suggesting that the in vitro assay preserved CD4 T cell plasticity (Geginat et al., 2014; Nakayamada et al., 2012; Weinmann, 2014; Lu et al., 2011; Tuzlak et al., 2021). Principal component analysis demonstrated high reproducibility of the assay with samples grouping tightly by genotype and stimulation (Fig. S4 B). STX11 abundance significantly increased after B cell interaction in SMARTA.WT CD4 T cells, suggesting functional involvement in T/B interaction (Fig. S4 C). Interestingly, a comparison of protein abundance following B cell interaction (d3) identified decreased protein levels for IL2ra, IL4r, and IL21r, but increased RAB27a in *Stx11*^*−/−*^ CD4 T cells in comparison with WT CD4 T cells after B cell interaction (Fig. 8, A and B). We confirmed reduced IL2ra surface expression by flow cytometry in *Stx11*^*−/−*^ CD4 T cells on d3 and d4 of T/B interaction and also identified reduced CD80 surface presentation by *Stx11*^*−/−*^ CD4 T cells (Fig. 8 C). Analysis of cytokines at d3 and d4 of T/B cell coculture demonstrated reduced IL-2 and IL-10 and increased IL-17 concentration in the supernatant of B cells co-cultured with *Stx11*^*−/−*^ CD4 T cells. Additionally, both CD4 T cells secreted comparable amounts of IFNγ, TNFα, IL-4, and IL-6 (Fig. 8 D). Since IL-21 cannot be reliably measured and protein levels of IL21r (MS data) were decreased in *Stx11*^*−/−*^ CD4 T cells, we investigated the effects of IL-21 on in vitro B cell differentiation by using IL21r-deficient B cells in the T/B cell interaction assay. Differentiation of IL21r-deficient B cells and WT B cells were comparable suggesting that impaired IL-21 signaling is not causative for the B cell defect observed in coculture with *Stx11*^*−/−*^ CD4 T cells, as IL-21 signaling seems to be redundant in this experimental setup (Fig. S4 D).

Taken together, impaired interaction between *Stx11*^*−/−*^ CD4 T and WT B cells results in impaired CD107a and CD40L mobilization and reduced IL-2 and IL-10 release by CD4 T cells leading to a secondary B cell defect.

## Discussion

STX11 is abundantly expressed in immune cells (Valdez et al., 1999; Prekeris et al., 2000; Zhang et al., 2008; Xie et al., 2009; Offenhauser et al., 2011; Dabrazhynetskaya et al., 2012) and required for lymphocyte cytotoxicity by CD8 T and NK cells, mediating fusion of lytic granules with the plasma membrane (Marshall et al., 2015; Arneson et al., 2007; Bryceson et al., 2007; D’Orlando et al., 2013). Our data demonstrate a previously unknown critical requirement for STX11 in CD4 T cell/B cell interaction, resulting in a secondary B cell defect. We excluded a general intrinsic role for STX11 in B cell differentiation by different experimental approaches in mice, like immunization with either TI or TD antigens, mixed BM chimeras, T cell transfers, and the antigen-specific in vitro T/B cell interaction assay. In line, in vitro stimulated human naive B cells from STX11-deficient (FHL-4) patients differentiated and underwent isotype switching, if optimal T cell help (mimicked by CD40L and IL-21 +/− IL4) was provided. However, FHL-4 patients with active HLH/hyperinflammation showed higher naive B cell frequencies. Additionally, the frequency of activated naive B cells was increased and the ratio of GC-independent DN (DN2) to GC-dependent (DN1) B cells was elevated, suggesting impaired GC-dependent differentiation. While the human data cannot exclude a role for IFNγ and hyperinflammation on the B cell phenotype, as postulated by Shim et al. (2023), detailed investigation of *Stx11*^*−/−*^ mice demonstrated impaired GC formation, reduced GC B cells and plasmablasts, and lower IgG levels with low avidity, independently of inflammatory conditions (Shim et al., 2023). Additionally, normal B cell differentiation in *Prf1-*deficient mice with severe hyperinflammation strengthens our conclusion of hyperinflammatory-independent B cell impairment in *Stx11* deficiency. Furthermore, CD4 T cell depletion in *Prf1*^*−/−*^ and WT mice resembled the B cell phenotype seen in STX11 deficiency, whereas CD4 T cell depletion in *Stx11*^*−/−*^ mice did not further exacerbate the secondary B cell defect. Thus, our data demonstrate a severe functional impairment of CD4 T cells lacking the SNARE protein STX11.

Tfh cells are the main provider of B cell help in the CD4 T cell compartment, determining the quality of antibody responses. For Tfh cell maturation, CD4 T cells are primed by dendritic cells (DCs), migrate to the T cell:B cell border, and interact with B cells for full differentiation (Crotty, 2011, 2015, 2019). We found normal Tfh frequencies in FHL-4 patients and in *Stx11*^*−/−*^ mice until d6 after LCMV infection. However, Tfh cells in *Stx11*^*−/−*^ mice did not further expand and showed reduced proliferative capacity when compared with *Prf1*^*−/−*^ or WT mice on d8 p.i. This might be explained by limited interaction with B cells in absence of STX11. Accordingly, we demonstrated a reduced percentage of *Stx11*^*−/−*^ CD4 T cells forming conjugates with B cells after 30 min of in vitro interaction.

Interestingly, in the T/B cell interaction assay *Stx11*^*−/−*^ CD4 T cells were able to activate naive WT B cells and induce proliferation but were impaired in promoting B cell expansion and maintenance. We further examined surface protein expression, since e.g., ICOS expression by CD4 T cells is important for B activation and differentiation (Grimbacher et al., 2003; Warnatz et al., 2006). Patients with deficiencies in ICOS have reduced B cell numbers and a lack of long-lived plasma cells (Kamperschroer et al., 2006, 2008; Schwartzberg et al., 2009; Crotty et al., 2003). The surface expression of costimulatory molecules PD1, ICOS, and the chemokine receptor CXCR5 were comparable between WT and *Stx11*^−/−^ CD4 T under these experimental conditions. Therefore, we investigated the regulated secretion of proteins by CD4 T cells to understand whether exocytosis of lysosomal vesicles is compromised in a similar way to lytic granule exocytosis in *Stx11*^−/−^ CD8 T cells (Arneson et al., 2007; Halimani et al., 2014). We found severe impairment of CD107a/LAMP1 mobilization to the surface in *Stx11*^−/−^ CD4 T cells. In line with this, a recent study of GC-Tfh and GC-Tfh–like cells identified transcriptional upregulation of pathways involved in membrane trafficking, like Golgi-ER transport, endosome vesicle vacuole organization, lipid metabolism and exocytosis/degranulation, and specifically demonstrated CD107a/LAMP1 upregulation in GC-Tfh cells (Yeh et al., 2022). Together, these data suggest a possible trafficking alteration for key molecules, which might be stored in intracellular vesicles. Such a key molecule essential for GC formation, isotype switching, and affinity maturation is CD40L, which is known to be stored in secretory lysosomes and exosomes (Casamayor-Palleja et al., 1995; Gilmour et al., 2003; Koguchi et al., 2007). About 50% of described CSR deficiencies are caused by impaired CD4 T cell help due to mutations in CD40L, which is expressed by CD4 T cells. CD40L deficiency results in a lack of GC and failure of isotype switching (Pietrella et al., 2004; Gilmour et al., 2003). This is a similar phenotype to the *Stx11*^*−/−*^ mice, wherein the CD40L is not mutated but mobilization to the surface of CD4 T cells is insufficient. Furthermore, we demonstrated by TIRF live-cell imaging that STX11 is transported by recycling endosomes (RAB11^+^) and MVBs (RAB7^+^) to the immunological synapse. This has also been described for cytotoxic granule fusion in human CD8 T cells (Halimani et al., 2014). In CD4 T cells, STX11^+^ hotspots might be crucial for CD40L^+^ vesicle/membrane fusion at the synapse. Impaired membrane trafficking might lead to the accumulation of RAB27a, which was identified by our proteomic analysis of SMARTA.*Stx11*^*−/−*^ CD4 T cells. RAB27a is involved in exosome release, which contains various proteins or microRNA (Ostrowski et al., 2010). It was recently described that microRNA release by Tfh cells via exosomes contributes to successful GC formation (Zeitrag et al., 2021), a function that is likely to be impaired in *Stx11* deficiency. Reduced fusion of recycling endosomes might explain the impaired CD40L mobilization to the surface seen in our assays. Additionally, since CD40L was also described to be directionally released in exosomes into the synaptic cleft and transferred to B cells as “help to go” (Gardell and Parker, 2017; Dustin, 2017; Saliba et al., 2019), impaired fusion of MVBs at the IS due to *Stx11* deficiency might also diminish the CD40L “help to go.” Fast transport and fusion of vesicles at the IS are essential since the interaction of Tfh and B cells in the GC is brief and is suggested to last ≤5 min (Shulman et al., 2013). Synergistic effects of CD40L and cytokines on B cell survival and differentiation have been described (Fluckiger et al., 1993). The involvement of STX11 in the transport of cytokines is likely, since other SNARE proteins like STX4 regulate TNFα secretion by macrophages and STX3 is important for IL-6 secretion by DCs (Pagan et al., 2003; Collins et al., 2015; Stanley and Lacy, 2010; Murray and Stow, 2014). Deficiencies in soluble factors like IL-21 are associated with impaired CD4 T cell help resulting in hypogammaglobulinemia (Salzer et al., 2014; Kotlarz et al., 2014). However, in vitro experiments excluded impaired IL-21 secretion by CD4 T cells because IL-21r–deficient B cells were able to differentiate and survive in the same way as WT B cells. Detailed analysis of cytokines in the supernatant at d3 and d4 of T/B interaction revealed reduced levels of IL-2 and IL-10. Additionally, proteomic results demonstrated reduced IL2ra, as well as reduced IL4r and IL21r, which might be the result of reduced IL-2 secretion and reduced autocrine stimulation by *Stx11*^*−/−*^ CD4 T cells in comparison with WT CD4 T cells. Furthermore, IL-2 signaling in CD4 T cells is known to attenuate IL-17 production (Kim et al., 2017; Laurence et al., 2007), which was higher in cocultures with *Stx11*^−/−^ CD4 T cells. Even though the effect of IL-2 and the expression of IL2ra on Tfh development is controversial (Ballesteros-Tato et al., 2012; Zhu et al., 2003), a dose-dependent positive effect on T/B cell interaction is possible, because low-intermediate expression of IL2ra (Pepper et al., 2011; Choi et al., 2011; Boyman and Sprent, 2012) and subsequent low-dose IL-2 (Ditoro et al., 2018) are reported for Tfh cells, whereas a high dose of IL-2 predominantly leads to Th1 responses by inhibiting BCL-6, but leaving T-BET expression unchanged. Therefore, the T-BET:BCL-6 ratio modulated in part by IL-2 can determine CD4 T cell fate and plasticity (Oestreich et al., 2012). IL2ra upregulation was also described in Tfh cells during booster responses (Merkenschlager et al., 2021). Reports of Tfh subsets, like Tfh1, with similar transcriptional regulation and cytokine production as Th1 cells and their importance in viral mouse infection models (Powell et al., 2019; Oestreich et al., 2012; Weinstein et al., 2018; Moon et al., 2015), further support a role of IL-2. Additionally, several reports demonstrate the promotion of GC B cells and plasmablasts (Johnson-Leger et al., 1998; Berglund et al., 2013) by IL-2. Furthermore, IL-10 produced by Tfh cells supports B cell differentiation into plasmablasts and isotype-switching (Defrance et al., 1992; Xin et al., 2018), which was also reduced in cocultures with *Stx11*^−/−^ CD4 T cells. Different studies investigating the release of CD40L, IL-2, IL-10, or IFNγ demonstrate a directional transport of these factors to the IS and release into the synaptic cleft (Huse et al., 2006; Boisvert et al., 2004), similar to cytotoxic granules in CD8 T cells. The impaired release of cytotoxic granules in STX11 deficiency is well described (de Saint Basile et al., 2010; D’Orlando et al., 2013; Chang et al., 2017), along with a positive effect of high-dose IL-2 on the exocytosis of cytotoxic granules by CD8 T cells in vitro (Bryceson et al., 2007).

Taken together, our data support a model in which CD40L, IL-2, and IL-10 might work synergistically to promote B cell differentiation and survival. Secretion of these proteins is decreased in *Stx11*^−/−^ CD4 T cells resulting in low expression of IL2ra, IL4ra, and IL21 and increased IL-17 release. Additionally, interaction between *Stx11*^−/−^ CD4 T cells and B cells might be shorter, since reduced percentages of *Stx11*^−/−^ CD4 cells involved in T/B conjugates were found. It is—however—challenging to discern the primary factor contributing to impaired CD4 T cell help, as the interaction between Tfh and B cells involves a multifactorial and multistep mechanism. Additionally, kinetics might influence the quality of CD4 T cell help.

In a clinical setting, the impaired GC formation and reduced isotype-switching in *Stx11* deficiency resulting in variable hypogammaglobulinemia, as discovered in this study, is expected to increase susceptibility to infections, which is also described for late-onset FHL-5 patients with hypomorphic mutations in *STXBP2,* a direct interaction partner of STX11 (Rohr et al., 2010; Meeths et al., 2010; Pagel et al., 2012; Esmaeilzadeh et al., 2015). Close monitoring of Ig levels and specific antibodies is therefore warranted in *Stx11* deficiency and Ig substitution should be initiated in FHL-4 infants in which abnormalities are detected.

## Materials and methods

### Patient material

The study was approved by the University of Freiburg’s Ethics Committee (143/12 and 40/08) and the Regional Ethics Review Board, Stockholm (2013/1723-31/4). STX11-deficient patients were selected independent of sex (male or female) or demographic origin; patients under 5 mo of age were excluded. Samples were not randomized or blinded since patient samples are rare and limited. Normal values (gray areas) for IgM and IgG serum levels (Oster, 2015) and B cell subpopulation depending on the age are included in the graphs (Piatosa et al., 2010). Healthy controls (independent of sex or demographic origin with an age between 9 and 13 years) were provided by the FREEZE Biobank, University Medical Center Freiburg. In accordance with the Declaration of Helsinki, informed consent was obtained from all patients or parents, as well as healthy controls.

### Cell lines

No cell lines were used in this study.

### Mice

Experiments were approved by the Regierungspraesidium Freiburg (G-17/15, G-18/160, X-15/05H, and X-20/04K). *Stx11*^−/−^ mice were generated by Udo zur Stadt by deletion of the only coding exon (D’Orlando et al., 2013; Kögl et al., 2013), backcrossed 10 times to C57BL/6J mice. *Prf1*^*−/−*^ (C57BL/6-*Prf1*^*tm1Sdz*^/J, RRID:IMSR_JAX:002407, obtained from Dr. Hengartner, Zurich), *Souris/Lyst*^−/−^ (C57BL/6-*Lyst*^*bg-Btlr*^/Mmcd, RRID:MMRRC_010470-UCD, purchased from Mutant Mouse Resource & Research Centers [MMRRC]), *Jinx* (C57BL/6J-*Unc13d*^Jinx^/Mmucd, RRID:MMRRC_016137-UCD, purchased from MMRRC and bred with *Unc13D*^WT/Jinx^ littermates), JHT (B6.129P2-^Igh-Jtm1Cgn^/J, RRID:IMSR_JAX:002438, received from emeritus professor H. Pircher), Tcra^−/−^ (B6.129S2- Tcra^tm1Mom^/J, RRID:IMSR_JAX:002115), *IL21r*^*−/−*^ (B6.129-Il21r^tm1Kopf^/J, RRID:IMSR_JAX:019115, received from Manfred Kopf, Institute for Molecular Health Sciences, ETH, University of Zurich, Switzerland), and WT mice (*Stx11*^*+/+*^ or *Stx11*^*+/−*^ littermates; background C57BL/6J or CD45.1 [B6.SJL-Ptprc^a^Pepc^b^/BoyJ, RRID:IMSR_JAX:002014]) or CD45.2 C57BL/6N (purchased from Janvier), were kept under specific-pathogen-free conditions. TCR-transgenic LCMV-GP_61-80_-specific SMARTA mice (B6.Cg-Ptprc^a^Pepc^b^Tg(TcrLCMV)1Aox/PpmJ, RRID:IMSR_JAX:030450) (Oxenius et al., 1998) were crossed with CD45.1^+^*Stx11*^*−/−*^ mice (background B6.SJL-Ptprc^a^ Pepc^b^/BoyJ). Mice were genotyped via PCR or flow cytometry for TCR transgenic mice. Experiments with mice were replicated two to three times, as indicated in the figure legends.

### BM chimera

BM chimera recipients were irradiated on two consecutive days (each 500 rad) and 10^7^ donor BM cells were injected i.v. Neomycin sulfate (2 mg/ml) was provided for 4 wk in drinking water.

### Transfer experiments

For AdTf, 2–6 × 10^6^ purified CD4 T cells (MojoSort mouse, BioLegend) from spleen/lymph nodes of LCMV-immune WT mice were injected i.v. SMARTA.WT or SMARTA.*Stx11*^*−/−*^ from naive mice were isolated and transferred into *Stx11*^*−/−*^ mice prior to LCMV infection.

### Viruses

Mice were infected with LCMV-WE, VSV, or VV_WR_ (provided by Dr. H. Pircher, Freiburg).

### CD4 or CD8 T cell depletion

T cell depletions were performed by i.p. antibody injection (anti-mouse CD4 [RRID:AB_10950382] or anti-mouse CD8 [RRID:AB_10950145]; BioXCell) on d3 and d1 before infection. LCMV was quantified using a focus-forming assay (Battegay et al., 1991).

### Immunization

Mice were immunized by i.p. injection of TI antigens (50 µg TNP-0.5-LPS or TNP-15-Ficoll in PBS; Biosearch Technologies) or TD antigens (100 µg TNP-17-KLH in Sigma-Aldrich Adjuvant System d0 and d28; Biosearch Technologies), and serum IgM/IgG3 antibody levels were measured.

### ELISAs

LCMV-NP–specific IgG was determined as described previously (Sommerstein et al., 2015; Schweier et al., 2019). Bulk IgM/IgG/TNP-specific plates were coated with AffinePure goat-anti-mouse IgM (RRID:AB_2338456), rabbit anti-mouse IgG (RRID:AB_2340040), or 10 µg/ml TNP-33-BSA (BiosearchTechnologies) in PBS as described (Schweier et al., 2019). Conjugated goat anti-mouse IgM (RRID:AB_2340070), anti-mouse IgG (RRID:AB_2340067; Jackson ImmunoResearch), or anti-mouse IgG3 (RRID:AB_2794588; Jackson ImmunoResearch) antibodies were used for detection. O-phenylendiamine dihydrochloride (Sigma-Aldrich) was added (5 min in the dark), stopped by 2 M H_2_SO_4_, and measured (absorbance 492 nm, TECANreader, Magellan2).

### Flow cytometry

Antibodies were purchased from Thermo Fisher Scientific (Invitrogen), BD Biosciences, Jackson ImmunoResearch, or BioLegend (Tables 1 and 2), and staining for flow cytometry was performed at 4°C in the dark (30 min, 4°C), except for Fas staining (20 min, 37°C). For intranuclear staining (Ki-67/Bcl-6), the FoxP3-Staining-Buffer Set (eBioscience) was used. Analyses were performed using the LSR Fortessa cytometer (BD Biosciences) and FlowJo software v8.8.7/v10.

**Table 1.** Anti-mouse antibodies for flow cytometry

Epitope	Clone	Fluorophore	Company	Catalog number	RRID
Ki-67 Ki-67	B56 16A8	PerCP-Cy5.5 AF700	BD Bioscience BioLegend	561284 652420	RRID:AB_10611574 RRID:AB_2564285
Bcl-6	BCL-DWN	PE	Thermo Fisher Scientific	12-5453-80	RRID:AB_2572620
IgM	RMM-1	AF488	BioLegend	406522	RRID:AB_2562859
IgD	11-26c.2a	BV510	BioLegend	405723	RRID:AB_2562742
Fas	Jo2	PE	BD Bioscience	554258	RRID:AB_395330
IgG		APC	SouthernBiotech	1033-31	RRID:AB_2794335
GL7	GL7	BV421	BioLegend	144614	RRID:AB_2563292
CD38	90	AF700	Invitrogen	56-0381-82	RRID:AB_657740
CD138	281-2	PE	BioLegend	142504	RRID:AB_10915989
B220 B220 B220	RA3-6B2	BV421 PerCp-Cy5-5 APC-Cy7	BioLegend	103251 103236 103224	RRID:AB_2562905 RRID:AB_893354 RRID:AB_313006
CD19	6D5	PeCy7	BioLegend	115520	RRID:AB_313655
CD43	S11	FITC	BioLegend	143203	RRID:AB_10959658
CD4 CD4 CD4	RM4-5 RM4-5 GK1.5	AF488 BV650 PerCp-Cy5-5	BioLegend	116004 100555 100434	RRID:AB_313689 RRID:AB_11126142 RRID:AB_893330
CXCR5 CXCR5	L138D7	PE BV421	BioLegend	145503 145511	RRID:AB_2561967 RRID:AB_2562127
PD1	29F.1A12		BioLegend	135225	RRID:AB_2563680
ICOS	C398:4a	PE-Cy7	BioLegend	313520	RRID:AB_10641839
CD62L	MEL-14	BV650	BioLegend	104453	RRID:AB_10641839
CD44	IM7	APC	BioLegend	103011	RRID:AB_312963
CD25/IL2ra	PC61	AF488	BioLegend	102017	RRID:AB_493334
CD80	16-10A1	PE-Cy7	BioLegend	104734	RRID:AB_2563112
CD107a	1D4B	BV421	BioLegend	121618	RRID:AB_2749905
CD40L	MR1	PE	BioLegend	106506	RRID:AB_313270
FasL	MFL3	PE	BioLegend	106606	RRID:AB_313279
CD45.1 CD45.1	A20	PerCP-Cy5.5 AF488	BioLegend	110727 110717	RRID:AB_893348 RRID:AB_492862
CD45.2	104	BV785	BioLegend	109839	RRID:AB_2562604
Live/dead		Zombie NIR dye	BioLegend	423106	

**Table 2.** Anti-human antibodies for flow cytometry

Epitope	Clone	Fluorophore	Company	Catalog number	RRID
CD11c	S-HCL-3	BV421	BioLegend	371512	RRID:AB_2650794
CD3	OKT3	SB436	Invitrogen	62-0037-42	RRID:AB_2734957
CD20	2H7	Pacific blue	BioLegend	302320	RRID:AB_493650
CD38	HIT2	Pacific blue	Exbio	PB-366-T100	RRID:AB_10736773
TCRgd	B1	BV480	BD Biosciences	566076	RRID:AB_10736773
CD19	HIB19	BV510	BioLegend	302242	RRID:AB_396135
CD8	RPA-T8	BV570	BioLegend	301038	RRID:AB_2563213
IgM	MHM-88	BV605	BioLegend	314524	RRID:AB_2562374
CD38	HB-7	BV650	BioLegend	356620	RRID:AB_2566232
IgG	G18-145	BV650	BD Biosciences	740596	RRID:AB_2740297
CD10	HI10a	BV711	BioLegend	312226	RRID:AB_2565876
IgD	IA6-2	BV711	BD Biosciences	740794	RRID:AB_2740457
CD4 CD4	SK3 RPA-T4	BV750 BV650	BD Biosciences BioLegend	566356 300536	RRID:AB_2744426 RRID:AB_2632791
CD27	L128	BV786	BD Biosciences	563327	RRID:AB_2744353
CD25	M-A251	Kiravia blue	BioLegend	356144	RRID:AB_2860935
CD95	DX2	PE	BD Biosciences	555674	RRID:AB_396027
CD21	Bu32	PE-Dazzle 594	BioLegend	354922	RRID:AB_2750243
IgG	Polyclonal	PerCP	Jackson ImmunoResearch	109-126-098	RRID:AB_2337686
IgD	IA6-2	PerCP-eFluor 710	Invitrogen	46-9868-42	RRID:AB_2573920
CXCR3	1C6/CXCR3	PE-Cy7	BD Bioscience	560831	RRID:AB_2033944
CXCR5	RF8B2	AF647	BD Biosciences	558113	RRID:AB_2737606
CD127	HIL-7R-M21	APC-R700	BD Biosciences	565185	RRID:AB_2739099
Zombie		Zombi NIR	BioLegend	423106	
CD45RA	HI100	APC-Fire750	BioLegend	304151	RRID:AB_2616714
CD107a	H4A3	PE	Invitrogen	555801	RRID:AB_396135

Single-cell suspensions from human B cells or PBMCs were stained for 20 min at room temperature (CXCR3 PRID:AB_2033944, CXCR5 PRID:AB_2737606, CD127 PRID:AB_2744353, TCRγδ PRID:AB_10736773) or for 15 min at 4°C with antibodies shown in Table 2. Samples were acquired using a Cytek Aurora (Cytek Biosciences) cytometer and analyzed with FlowJo software v10.7.1 (BD Biosciences).

### Histology

#### PNA/B220

Sections from embedded (Tissue-Tek compound; Sakura Finetek) snap-frozen spleens were sliced using Cryostat (CM 1850; Leica) and dried on SuperFrost Plus slides (R. Langerenbrinck GmbH) and were fixed in methanol (−15°C, 20 min), blocked with 5% mouse/rat serum, stained with fluorochrome-labeled peanut-agglutinin—Cy3 (RRID:AB_2336640; #CL-1073-1; Vector Laboratories) in PBS (+Ca2/Mg2) for 2 h at room temperature and rat anti-mouse B220-AF488 (RRID:AB_389308; BioLegend) in PBS (without Ca2/Mg2) at 4°C overnight.

#### Bcl-6/B220/CD4

Spleens were fixed in 4% formaldehyde at 4°C for 30 min. Afterward water was removed by incubation in 30% sucrose in PBS at 4°C overnight. Spleens were embedded (Tissue-Tek compound; Sakura Finetek) and snap-frozen. Organs were sliced using Cryostat (CM1850; Leica) and dried on SuperFrost Plus slides (R. Langerenbrinck GmbH). Blocking was done in 0.3% Triton X-100, 1% BSA in PBS + 10% Mouse serum/Rat serum for 2 h, room temperature. Antibodies were added in PBS + 0.3% Triton x-100; anti-mouse BCL6 PE (clone: BCL-DWN, RRID:AB_2572620; BD Bioscience), anti-mouse B220-AF488 (RRID:AB_389308; BioLegend), and anti-mouse CD4-AF647 (RRID:AB_389324; BioLegend) and incubated overnight at 4°C. Images were collected with a ZEISS Imaging Axioplan 2 microscope (Axiovision software v4.8.2.0).

### In vitro human B cell stimulation

PBMCs were purified from blood by density gradient centrifugation. Naive CD27^−^ B cells were isolated with the EasySep Human Naive B cell Isolation Kit (STEMCELL Technologies) following the manufacturer’s instructions. Isolated naive B cells were plated in supplemented IMDM (Thermo Fisher Scientific) as described previously (Warnatz et al., 2009) at a concentration of 0.15 × 10^6^ cells/ml. Cells were stimulated with trimeric CD40L and IL-21 as described (Warnatz et al., 2009) in the presence or absence of IL-4 (25 ng/ml; Immunotools).

### STX11 western blot

#### Mouse

Purified murine CD4 T and B cells were stimulated with anti-CD3/CD28 activator dynabeads (11456D; Thermo Fisher Scientific) for 72 h or anti-mouse IgM for 30 min at 37°C. Cell lysates (5 µg) were loaded on Nu PAGE 10% Bis-Tris gels (Thermo Fisher Scientific), transferred to nitrocellulose membranes, and blocked (5% nonfat dry milk, 20 mM Tris, 0.15 M NaCl, and 0.05% Tween20). Anti-mouse STX11 antibody (RRID:AB_10639254; Synaptic System) was added and incubated overnight at 4°C, followed by HRP secondary antibody (RRID:AB_228307; Thermo Fisher Scientific) labeling. The blot was developed by chemiluminescence (Thermo Fisher Scientific) and imaged (FlourChem E system; BioLabTec). Anti-β actin antibody (RRID:AB_476697; Sigma-Aldrich) was used after stripping.

#### Human

10^6^ cells were lysed in 20 µl radioimmunoprecipitation assay buffer (50 mM Tris-HCl, 150 mM NaCl, 1 mM EDTA, 0.5% NaDeoxycholate, 0.05% SDS, 1% Igepal supplemented with cOmplete protease inhibitor cocktail [Roche; Roche Diagnostics]). The protein contents of the lysates were measured by Bradford assay (Bio-Rad Laboratories). Lysates (15 µg protein per lane) were transferred onto nitrocellulose membranes. Membranes were blocked with 5 % low-fat milk in PBS-Tween 20 (0.05 % vol/vol) for 1 h at room temperature. For the detection of STX11, membranes were incubated with rabbit anti-human STX11 antibody (5413, homemade and provided by G.M. Griffiths, Cambridge Institute for Medical Research, The Keith Peters Building, Cambridge Biomedical Campus, Cambridge, UK) overnight at 4°C and with HRP-conjugated rabbit anti-mouse antibody (RRID:AB_2617138; Dako) for 1 h at room temperature. Human β-actin was detected as loading control by incubation of the membrane at room temperature for 2 h with HRP-labeled rabbit anti-β-actin antibody (RRID:AB_2883836; Cell Signaling Technology Europe). Western blots were developed with an enhanced chemiluminescence (ECL) system. Protein bands were detected and analyzed with the help of an ECL ChemoStar Imager HR 6.0 and the software LabImage 1D (Science Instruments GmbH).

### CD4 T cell culture

#### Human

Naive CD4 T cells of healthy controls were purified using MojoSort (BioLegend), stimulated at a concentration of 5 µl/10^6^ cells/ml anti-CD3/CD28/CD2 beads (STEMCELL Technologies SARL) in X-VIVO 15 medium (Lonza Sales Ltd.) + 5% antibody (AB) serum (Sigma-Aldrich) and 100 ng/ml rhIL-12p70 (PeproTech) until further use.

#### Mouse

1.6 × 10^6^ purified CD4 T cells from SMARTA mice were stimulated with 1 µg/ml plate-bound anti-mouse CD3 (RRID:AB_395697; BD Bioscience) and 2 µg/ml soluble anti-mouse CD28 antibody (RRID:AB_1107624; BioXCell) in 24-well plates. On d2, cells were transferred into uncoated wells and soluble CD28 antibody was added to new media until d4.

### CRISPR-Cas9 approach

Human T cells were used on d3 of culture. Six guide RNAs (gRNA) were designed to disrupt the human *STX11* gene using CHOPCHOP. RNPs were preassembled at a 1:3 M ratio by incubating 18.3 pmol (3 µg) of Cas9 protein (IDT) with 55 pmol of gRNA (Biolegio; Table 3) for 10 min at room temperature. Nucleofections of 10^6^ naive CD4 T cells (P3-EO115) with a combination of up to six RNPs were performed using a 4D-Nucleofector (Lonza).

**Table 3.** gRNA sequences, related to experimental procedures

Cleavage site	Guide ID	Direction	Sequences (5′-3′)	PAM
*STX11* intron 1	i1	Forward	5′-TGC​ACT​TAT​TGC​CCA​CA^**↓**^CCG-3′	AGG
*STX11* exon 2	e1	Forward	5′-TGA​CCA​GCA​GTT​CCC​AG^**↓**^ACG-3′	GGG
*STX11* exon 2	e2	Reverse	5′-TCG​AAC​ACG​ATG​TCC​TC ^**↓**^GTG-3′	GGG
*STX11* exon 2	e3	Reverse	5′-CTT​TCC​CAG​CCG​CTT​CA^**↓**^CGT-3′	CGG
*STX11* exon 2	e4	Forward	5′-AGT​CTC​GGG​CGA​CCA​GA^**↓**^TCG-3′	AGG
*STX11* exon 2	e5	Reverse	5′-GTT​CTT​CTC​CTC​GTA​CT^**↓**^GCA-3′	CGG

### Antigen-specific in vitro T/B cell interaction assay

Stimulated mouse T cells were used for experiments on d6. Sorted CFSE-labeled, naive WT C57BL/6N mice B cells were pulsed with 5 × 10^−7^M LCMV-GP_61-80_ peptide (PolyPeptide) for 2 h or left unpulsed (37°C). 4 × 10^4^ washed B cells were mixed with 2 × 10^4^ preactivated LCMV-GP_61-80_-specific SMARTA CD4 T cells in round-bottom 96-well plates.

### T/B conjugates

B cells were isolated from naive WT C57BL/6N mice using Pan-B Cell-Isolation-Kits (480052; BioLegend), stained with CFSE, labeled, and stimulated overnight with 1 µg/ml LPS (Sigma-Aldrich) in IMDM (GibcoFisher Scientific) +10% FCS (anprotec) + GlutaMax (GibcoFisher Scientific) + β-ME (GibcoFisher Scientific). Cells were washed and either pulsed with 10^−6^M LCMV-GP_61-80_ peptide (PolyPeptide) or left unpulsed for 2 h, 37°C, 5% CO_2_. 10^6^ cells/ml LPS-activated/CFSE-labeled B cells were incubated with 2.5×10^6^/ml d7 cultured/Cell Trace Violet (C34557; Thermo Fisher Scientific) CD4 T cells for 30 min. Cells were centrifuged 6 min, 400 rpm, prior to incubation.

### Immunofluorescence

#### Conjugates

Conjugates were prepared as described above without CFSE/Cell Trace Violet labeling. After 10 min, 37°C, 5% CO_2_ incubation, serum-free IMDM was added (3:1) and 50 µl cells were plated on multiwall slides for another 25 min, 37°C, 5% CO_2_. Cells were fixed with 4% paraformaldehyde (15710-S; Electron Microscopy Systems) and incubated in quenching solution (50 mM ammonium chloride in 1X PBS) (15 min at room temperature) and rinsed twice in PBS. Permeabilization was done with 0.2% Saponin in PBS (5 min, room temperature) followed by washing and blocking for 30 min in blocking buffer (1X PBS, 1% BSA, 0.2% Saponin). Cells were stained with anti-LAMP1-PE (RRID:AB_2134487; BioLegend), B220-AF488 (RRID:AB_389308; BioLegend), and anti-mouse CD4-AF647 (RRID:AB_389324; BioLegend). Slides were imaged with LSM880, Zeiss.

#### Plasmids

To generate the pMax-m*Stx11*-mNeonGreen vector, the mouse *Stx11* gene sequence was amplified from the pMax-m*Stx11*-IRES-TagBFP2 background vector. The forward primer (5′-ATG​TAT​AGA​ATT​CGC​CGC​CAC​CAT​GAA​GGA​TCG​GCT​T-3′) and reverse primer (5′-ATG​TAT​ACG​CGG​ATC​CGT​TGA​CAC​AGG​GAC​AAC​AGA​A-3′) were used, introducing EcoRI and BamHI restriction sites. Subsequently, the pMax-mNeonGreen plasmid was digested with EcoRI and BamHI restriction enzymes to serve as the background vector. The amplified m*Stx11* product was then ligated into the digested pMax-mNeonGreen vector, resulting in the construction of the pMax-m *Stx11*-mNeonGreen vector. The final vector was confirmed through plasmid sequencing using the respective forward and reverse primers by Microsynth Seqlab. The *Rab* constructs were created by inserting mouse *Rab11a* or *Rab7a* sequences into the pmCherry-C1 vector backbone. In both fusion proteins, mCherry is located at the N-terminus.

#### SIM

5 × 10^6^ d5 effector Stx11 KO CD4 T cells were cotransfected with *Stx11*-mNeonGreen (3 µg) and either *Rab11*-mCherry (3 µg) or *Rab7*-mCherry plasmids (3 µg) using the mouse T cell Nucleofector kit (LONZA). The transfection procedure was followed according to the manufacturer’s instructions. Cells were incubated in the transfection medium provided by LONZA overnight at 37°C, 5% CO_2_, allowing for the generation of overexpressed proteins after electroporation before use in experiments. The next day, cells were collected from the culture, resuspended in the culture medium, and placed on anti-CD3 antibody-coated (RRID:AB_394591) coverslips to form synapses for 30 min at 37°C in a 5% CO_2_ incubator before fixation with cold 4% PFA. Fixed cells were washed with Dulbecco’s Balanced Salt Solution and underwent immunostaining with biotinylated anti-CD40L (RRID:AB_313268; BioLegend) and anti-LAMP-1-Alexa647 (RRID:AB_571990; BioLegend) antibody. Cells were permeabilized with 0.1% Triton, followed by 2% BSA blocking for 30 min and a 1 h incubation at room temperature with the primary antibody. Finally, cells were stained with streptavidin-Alexa647 (Thermo Fisher Scientific) for 45 min. Subsequently, cells were mounted with a Mowiol-based mounting medium and observed using a SIM microscope (Elyra PS.1; Zeiss). Post-processed images were analyzed to achieve a resolution of 100 nm in the x and y dimensions and 200 nm in the z dimension.

#### Live-cell TIRF imaging

d5 effector WT CD4 T cells were electroporated with *Stx11*-mNeonGreen and either *Rab11*-mCherry or *Rab7*-mCherry plasmids to visualize STX11^+^ vesicle transportation and their fusion at the synapse by TIRF microscopy (TIRFM). The TIRFM setup was from Olympus (Olympus Europa SE and Co KG) and equipped with a solid-state laser (85 YCA) emitting at 561 nm (Melles-Griot). T cells (0.2 × 10^6^ cells) were resuspended in 30 µl of extracellular buffer (2 mM Hepes, 140 mM NaCl, 4.5 mM KCl, and 2 mM MgCl_2_) containing no Ca^2+^ and allowed to settle for 1 min on anti-CD3 antibody-coated (RRID:AB_394591) coverslips (30 µg/ml). Subsequently, cells were perfused with extracellular buffer containing 10 mM Ca^2+^ to maximize vesicle fusion probability. Cells were imaged for 8 min at room temperature and excited at 488 and 561 nm sequentially for vesicle polarization and fusion analysis using an Olympus 100× Plan-Apochromat objective (NA 1.45). The acquisition frequency was 10 Hz and the exposure time was 50 ms. Images were captured with a QuantEM512SC camera (Photometrics) using Visiview software (Visitron GmbH). Fusion of vesicles was analyzed using ImageJ with the plugin Time Series Analyzer V2.0. A sudden drop in fluorescence within 300 ms (three acquisition frames) was defined as fusion.

### In vitro CD107a, CD40L mobilization assay

#### Human

CD4 T cell cultures were restimulated at d10 for 3.5 h, 37°C, 5% CO_2_ with 2 µg/ml plate-bound anti-human CD3 (RRID:AB_571927; BioLegend) and anti-human CD28 (RRID:AB_314303; BioLegend) or 50 ng/ml PMA (Sigma-Aldrich) + 1 ug/ml Iono (Sigma-Aldrich) in X-VIVO 15 medium (Lonza Sales Ltd.) + 5% AB serum (Sigma-Aldrich). Anti-CD107a PE (RRID:AB_396135; BD Bioscience) was added to the medium prior to incubation. Cells were stained with anti-human CD4 and ZombiNIR and analyzed by flow cytometry.

#### Mouse

Preactivated T cells (d6) or in vivo activated (d12 p.i.) were restimulated with 50 ng/ml PMA (Sigma-Aldrich) + 1 ug/ml Iono (Sigma-Aldrich) for 30 min or 15 min. CD107a mobilization was additionally assessed after being restimulated with 10 µg/ml plate-bound anti-mouse CD3 (RRID:AB_395697) for 2 h. Anti-mouse CD107a (RRID:AB_2749905) and/or CD40L (RRID:AB_313270) antibodies were added prior to incubation time. Cells were stained with anti-mouse CD4 (RRID:AB_11126142) and ZombiNIR was analyzed by flow cytometry.

### Cytokine analysis/LEGENDplex

Cells were cocultured for 3 or 4 days at 37°C and 5% CO_2_ and culture supernatants were collected. Levels of various biomarkers were determined using the LEGENDplex Mouse B cell Panel (13-plex) kit according to the manufacturer’s instructions. In brief, standard and diluted or undiluted supernatant samples were incubated with antibody-conjugated beads, which capture their specific target analytes. Beads can be differentiated by size and internal fluorescence. After washing, biotinylated detection antibodies were added to bind the analytes captured by the beads. Finally, streptavidin-phycoerythrin was added to bind the biotinylated detection antibodies and data were collected using a BD LSR Fortessa II flow cytometer. Data were analyzed based on a standard curve recorded for each factor and run using the online software provided by the manufacturer.

### Proteomics and analysis

T/B cell interaction assay was scaled up and performed as described above; CD4 T cells were sorted at d0 (pre) or d3 (after) of T cell/B cell coculture (CD4^+^CD45.1^+^CFSE^−^B220^−^,ZombiNIR^−^). Cells were pelleted and frozen at −80.

### Tandem mass tag (TMT) protein analysis

16 samples (four replicates of each condition [four conditions: CD4-cultured WT and CD4-cultured STX11^−/−^ cells before T/B assay as well as WT and STX11^−/−^ CD4 T cells at d3 of coculture after cell sorting]) were analyzed in a TMT-label-based proteome comparison. Pelleted cells were resuspended in STRAP lysis buffer (final concentration: 5% SDS, 50 mM triethyl ammonium bicarbonate [TEAB], pH 7.5) and centrifuged at maximum speed for 8 min. The supernatant was taken and proteins were reduced using 5 mM tris (2-carboxyethyl) phophine hydrochloride (75259; Sigma-Aldrich) for 10 min at 95°C and alkylated using 10 mM 2-iodoacetamide (I1149; Sigma-Aldrich) for 20 min at room temperature in the dark. The following steps were performed using S-Trap micro filters (Protifi) following the manufacturer’s procedure. Briefly, a final concentration of 1.2% phosphoric acid and then six volumes of binding buffer (90% methanol; 100 mM TEAB; pH 7.1) were added. After gentle mixing, the protein solution was loaded to an S-Trap filter and spun at 2,000 rpm for 0.5–1 min. The filter was washed three times using 150 μl of binding buffer. Sequencing-grade trypsin (Promega, 1:25 enzyme:protein ratio) diluted in 20 µl digestion buffer (50 mM TEAB) was added into the filter and digested at 47°C for 1 h. To elute peptides, three step-wise buffers were applied: (1) 40 μl 50 mM TEAB, (2) 40 µl 0.2% formic acid in H_2_O, and (3) 50% acetonitrile and 0.2% formic acid in H_2_O. The peptide solution was combined and dried in a SpeedVac.

The peptide concentration was measured using BCA (#23225; Pierce) and 16 µg of each sample was transferred to a fresh microreaction tube. 0.15 M Hepes pH 8.0 was added. Samples were labeled using TMT-16-plex (Thermo Fisher Scientific) (Thompson et al., 2003). After labeling, all samples were combined and 100 µg were fractionated by high pH reversed-phase chromatography (XBridge C18 column, 150 × 1 mm column containing 3.5 µm particles [Waters]). An increasing linear gradient of acetonitrile from 10 to 45% over 45 min at a flowrate of 42 µl/min was applied using an Agilent 1100 HPLC system. 48 fractions were collected and concatenated into 16 fractions, which were vacuum-concentrated until dryness and stored at −80°C until liquid chromatography-MS/MS analysis.

800 ng of peptides were analyzed on a Q-Exactive Plus mass spectrometer (Thermo Fisher Scientific) coupled to an EASY-nLCTM 1000 UHPLC system (Thermo Fisher Scientific).

The column setup consisted of an Acclaim PepMap 100 C18 precolumn (#164946; Thermo Fisher Scientific) and a 200 cm µPac GEN1 analytical column (PharmaFluidics, 55250315018210) coupled to a Nanospray Flex ion source (#ES071; Thermo Fisher Scientific) and a fused silica emitter (TIP1002005-5; MS Wil). For peptide separation, a linear gradient of increasing buffer B (0.1% formic acid in 80% acetonitrile, Fluka) was applied, ranging from 5 to 50% buffer B over the first 80 min and from 50 to 100% buffer B in the subsequent 40 min (120 min separating gradient length). Peptides were analyzed in data-dependent acquisition mode. Survey scans were performed at 70,000 resolution, an automatic gain control (AGC) target of 3e6, and a maximum injection time of 50 ms followed by targeting the top 10 precursor ions for fragmentation scans at 17,500 resolution with 1.6 m/z isolation windows, a normalized collision energy of 30, and a dynamic exclusion time of 35 s. For all MS2 scans, the intensity threshold was set to 1e5, the AGC to 1e4, and the maximum injection time to 80 ms.

Raw data were analyzed with MaxQuant (v 1.6.14.0) with the built-in Andromeda peptide search engine (Cox and Mann, 2008) allowing two missed cleavage sites, no variable modifications, carbamidomethylation of cysteines as fixed modification, preimplantation factor was set to 0.75, and 16 plex TMT as the isobaric label. The mouse-EBI-reference database was downloaded from https://www.ebi.ac.uk/ on October 14, 2022. Only unique peptides were used for quantification.

Data were normalized on peptide level by equalizing the medians across all the channels and MS runs using the MSstatsTMT package (v. 1.8.2) in R (v. 4.0.3). Subsequent analysis was carried out in Perseus software (version 2.0.10) (Tyanova et al., 2016). Data were first filtered to remove matches to the reverse database, common contaminants, and matches based on modified peptides only. Additional filtering removed proteins quantified by only a single peptide. To obtain P values for differential protein expression a two-tailed *t* test was used. Significance was assessed using a permutation-based (250 permutations) estimated false discovery rate (FDR) of < 0.05 and an S0 parameter of 0.1. Principal component analysis (PCA) and hierarchical clustering were also carried out in Perseus.

### Statistical tests

Data were analyzed using SigmaPlot 9.0 or GraphPad Prism 8. Significant differences were evaluated using unpaired Student’s *t* test or Mann–Whitney *U* test.

### Online supplemental material

Two tables, four figures, and two videos are provided. Table S1 provides information about age, treatment, and description of the STX11 mutation for all FHL-4 patients analyzed for IgM/IgG serum levels and/or B cell phenotyping. Table S2 lists proteins for which abandunce is significantly altered in STX11-deficient versus WT CD4 T cells after T-B cell interaction identified by MS. Fig. S1 shows the gating strategies for the human B cell phenotyping, detailed B cell phenotyping of six FHL-4 patients and healthy controls, and steady-state B cell phenotyping for different organs in *Stx11*-deficient mice. Fig. S2 shows body weight of CD8 T cell–depleted *Stx11*-deficient and WT mice and immunohistological analysis of GC in spleens after VV and VSV formation; STX11 protein expression in mouse CD4 and B cells; gating strategy for human Tfh cells; and information on CD4 T cell compartment in mice and correlation between transferred WT CD4 T cells and GC B cells. Fig. S3 shows the gating strategy for t-SNE analysis and cluster identification for t-SNE analysis. Fig. S4 shows proteome alterations and reproducibility and IL-21 exclusion by T/B interaction with IL21r-deficient B cells. Video 1 demonstrates localization at the IS of STX11 and RAB11 and Video 2 shows localization of STX11 and RAB7 in WT CD4 T cells.

## Supplementary Material

Table S1 shows *STX11*-deficient/FHL-4 patient information.

Table S2 lists proteins for which abandunce is significantly altered in STX11-deficient versus WT CD4 T cells after T-B cell interaction identified by MS.

SourceData FS2 is the source file for Fig. S2.

SourceData F7 is the source file for Fig. 7.

## Data Availability

Mass spectrometry raw data, underlying Fig. 8 and Fig. S4, have been deposited at the ProteomeXchange Consortium (http://proteomecentral.proteomexchange.org) under the accession number PXD050263. Furthermore, all MS proteomics datasets used and/or analyzed during this study are available online at the MassIVE repository (http://massive.ucsd.edu/; dataset identifier: MSV000094210). This study did not generate new code for analysis.
