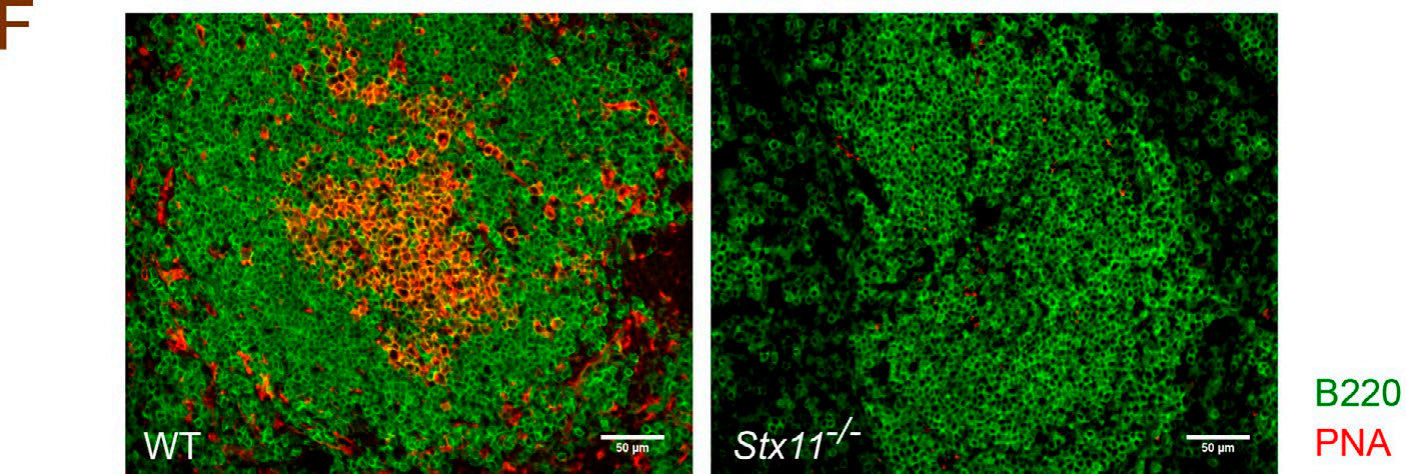# Correction: Patients and mice with deficiency in the SNARE protein SYNTAXIN-11 have a secondary B cell defect

**DOI:** 10.1084/jem.2022112205142024c

**Published:** 2024-05-21

**Authors:** Tamara Kögl, Hsin-Fang Chang, Julian Staniek, Samuel C.C. Chiang, Gudrun Thoulass, Jessica Lao, Kristoffer Weißert, Viviane Dettmer-Monaco, Kerstin Geiger, Paul T. Manna, Vivien Beziat, Mana Momenilandi, Szu-Min Tu, Selina J. Keppler, Varsha Pattu, Philipp Wolf, Laurence Kupferschmid, Stefan Tholen, Laura E. Covill, Karolina Ebert, Tobias Straub, Miriam Groß, Ruth Gather, Helena Engel, Ulrich Salzer, Christoph Schell, Sarah Maier, Kai Lehmberg, Tatjana I. Cornu, Hanspeter Pircher, Mohammad Shahrooei, Nima Parvaneh, Roland Elling, Marta Rizzi, Yenan T. Bryceson, Stephan Ehl, Peter Aichele, Sandra Ammann

Vol. 221, No. 7 | https://doi.org/10.1084/jem.20221122 | May 9, 2024

The authors regret that the “WT” and “*Stx11*^*−/−*^” labels were not visible in the immunohistology images of the originally published Fig. 1 F. The corrected panel is shown here. This correction does not change the original conclusions of the article, and the figure legend remains unchanged. The error appears in PDFs downloaded before May 14, 2024.

**Figure 1:**