# Supplementary material for: Patients and mice with deficiency in the SNARE protein SYNTAXIN-11 have a secondary B cell defect
Source: J Exp Med. 2024 May 9;221(7):e20221122. doi: 10.1084/jem.20221122 (PMC11082451; doi:10.1084/jem.20221122)
Supplement: Table S1 — shows STX11-deficient/FHL-4 patient information. [file JEM_20221122_TableS1.docx]

|  | **age at analysis** | **IgG** | **IgA** | **IgM** | **detailed B cell stain** | **IVIG** | **active**  **HLH** | **other treatment** | **mutation** |
| --- | --- | --- | --- | --- | --- | --- | --- | --- | --- |
| 1 | 9 years | 3.21 | 1.4 | 3.6 | x | no | yes | no | homozygote: c.369-370delAG/c.374_376delCGC; p.Val124*X60 |
| 2 | 2 years | 4.1 | 0.64 | 1.11 | x | no | yes | 2 days dexamethasone | homozygote:  AL135917:g.25561_44749del, |
| 3 | 8 months | 4.09 | 0.37 | 0.46 | nd | no | yes | no | homozygote:  c.[369_370del;374_376del]; p.Val124Glyfs*60 |
| 4 | 2.2 years | 2.59 | 0.54 | <0.25 | nd | no | yes | no | homozygote: c.369_370delAG/c.374_376delCGC  p.Val124fsX60 |
| 5 | 16 months | 6.24 | 0.51 | 1.23 | nd | no | yes | no | homozygote: c.369_370delAG/c.374_376delCGC p.Val124fsX60 |
| 6 | 11 years  5 months | 7.87 | 0.958 | 1.07 | nd | no | Yes | no | homozygote:  chr6:144498027-144517211 deletion |
| 7 | 5 months | IVIG | IVIG | IVIG | x | yes | yes | no | homozygote:  c.369_376delinsTGG p.Val124fsX60 |
| 8 | 6 years | 3.34 | <0.26 | 0.588 | nd | no | yes | no | homozygote:  c.369_376delinsTGG;  p.Val124fsX60 |
| 9 | 10 years | 4.24 | 0.428 | 0.571 | x | no | yes | no | homozygote:   c.290delG;  p.Gly97AlafsX11 |
| 10 | 1 year  7 months | 7.64 | 0.1 | 0.341* | x | no | yes | no | homozygote:   c.369_370delAG  p.Val124GlyfsX61 |
| 11 | 7 years  3 months | 13.55 | 1.935 | 1.274* | nd | no | yes | no | homozygote:  chr6:144498027-144517211 deletion |
| 12 | 15 years | nd | nd | nd | nd | no | asymptomatic | no | homozygote:  deletion of exon 2 in STX11 (144507765-144508658) |
| 13 | 5 yers | nd | nd | nd | x | no | yes | HLH-2004 | homozygote:  c.675_679del:p.E226Lfs*127 CGAGC – frameshift deletion |

Table 1: *STX11*-deficient/FHL-4 patient information

**nd** = not done, ***** = control measurements with high background; IVIG, i.v. Ig
