# Supplementary material for: Patients and mice with deficiency in the SNARE protein SYNTAXIN-11 have a secondary B cell defect
Source: J Exp Med. 2024 May 9;221(7):e20221122. doi: 10.1084/jem.20221122 (PMC11082451; doi:10.1084/jem.20221122)
Supplement: Table S2 — lists proteins for which abandunce is significantly altered in STX11-deficient versus WT CD4 T cells after T-B cell interaction identified by MS. [file JEM_20221122_TableS2.docx]

Table 2: significant proteins STX11 vs WT post T-B cell interaction

| Significant | minusLOG(P-value) | Difference (log2 fold change) | Gene.names | Protein.IDs | Peptides | Razor...unique.peptides | Unique.peptides |
| --- | --- | --- | --- | --- | --- | --- | --- |
| + | 1.680216815 | -0.651867628 | Mllt3 | A2AM29 | 2 | 2 | 2 |
| + | 3.198726533 | -0.399541527 | Slc43a3 | A2AVZ9 | 2 | 2 | 2 |
| + | 1.501864053 | -0.551500499 | Batf | O35284 | 2 | 2 | 2 |
| + | 1.51936929 | 0.547204733 | Bcl2l11 | O54918 | 2 | 2 | 2 |
| + | 1.919740058 | 0.510734677 | Zbtb7a | O88939 | 2 | 2 | 2 |
| + | 1.611119842 | 0.645499296 | Cox7c | P17665 | 2 | 2 | 2 |
| + | 2.084673999 | 0.54137817 | Des | P31001 | 8 | 2 | 2 |
| + | 3.908330201 | -0.617892355 | Fosl2 | P47930 | 2 | 2 | 2 |
| + | 1.50904371 | 0.496277288 | Cox7b | P56393 | 2 | 2 | 2 |
| + | 2.588609748 | -0.383138597 | Gfi1 | P70338 | 2 | 2 | 2 |
| + | 1.827404884 | -0.463076591 | Eps8 | Q08509 | 2 | 2 | 2 |
| + | 1.467483645 | 0.641406953 | Zfp607;C030039L03Rik;1700049G17Rik;Zfp780b | Q3TQG9;G3X9H3;Q3UVF6;E9Q2S6 | 2 | 2 | 2 |
| + | 1.95059147 | 0.44009757 | Cd96 | Q3U0X8 | 2 | 2 | 2 |
| + | 2.406053905 | -0.443349719 | Bahd1 | Q497V6 | 2 | 2 | 2 |
| + | 1.665911448 | -0.427249789 | Srek1ip1 | Q4V9W2 | 2 | 2 | 2 |
| + | 2.193577076 | -0.503571749 | Nkapl;Nkap | Q5SZT7;Q9D0F4 | 2 | 2 | 2 |
| + | 2.876709301 | -0.354486763 | Gpr65 | Q61038 | 2 | 2 | 2 |
| + | 2.537201362 | -0.370378941 | Rps27l | Q6ZWY3 | 4 | 2 | 2 |
| + | 1.480195775 | -0.655207276 | Znrf2 | Q71FD5 | 2 | 2 | 2 |
| + | 2.445369794 | -0.348677576 | Cpeb4 | Q7TN98 | 2 | 2 | 2 |
| + | 2.848004563 | -0.643116146 | Actbl2 | Q8BFZ3 | 12 | 2 | 2 |
| + | 1.894125337 | -0.354308724 | Eif1ax | Q8BMJ3 | 9 | 9 | 2 |
| + | 2.690771339 | 0.451483488 | Pqlc3 | Q8C6U2 | 2 | 2 | 2 |
| + | 2.430652241 | 0.36394912 | H2-Q4 | Q8HWB2 | 9 | 4 | 2 |
| + | 1.587202708 | -0.468611807 | Smyd2 | Q8R5A0 | 2 | 2 | 2 |
| + | 3.698102457 | -0.801585674 | Klhdc3 | Q8VEM9 | 2 | 2 | 2 |
| + | 2.735550045 | -0.361384392 | Itm2c | Q91VK4 | 2 | 2 | 2 |
| + | 2.63005631 | -0.390127659 | Polr3d | Q91WD1 | 2 | 2 | 2 |
| + | 1.731109346 | 0.451117337 | Mrpl51 | Q9CPY1 | 2 | 2 | 2 |
| + | 1.459714684 | 0.547252238 | Ndufb4 | Q9CQC7 | 2 | 2 | 2 |
| + | 2.673710992 | -0.580268681 | Cldnd1 | Q9CQX5 | 2 | 2 | 2 |
| + | 2.608604525 | -0.347396642 | Fam32a | Q9CR80 | 2 | 2 | 2 |
| + | 1.723220372 | 0.41933465 | Mrps25 | Q9D125 | 2 | 2 | 2 |
| + | 1.801423752 | -0.394215435 | Aar2 | Q9D2V5 | 2 | 2 | 2 |
| + | 2.111092139 | 0.465060442 | Mtfmt | Q9D799 | 2 | 2 | 2 |
| + | 2.261901776 | -0.607582569 | Fxr2 | Q9WVR4 | 5 | 2 | 2 |
| + | 2.005593739 | -0.407926083 | Arid4b | A2CG63 | 3 | 3 | 3 |
| + | 3.199806813 | 0.333500624 | Acad12;Acad10 | D3Z7X0;Q8K370 | 3 | 3 | 3 |
| + | 1.59003507 | -0.604364395 | Wdfy1 | E9Q4P1 | 3 | 3 | 3 |
| + | 2.110960125 | -0.339819759 | Plekha5 | E9Q6H8 | 3 | 3 | 3 |
| + | 2.609259839 | -0.362042308 | Abcb1a | P21447 | 16 | 3 | 3 |
| + | 1.064715335 | 2.275859714 | Acp2 | P24638 | 3 | 3 | 3 |
| + | 3.043242462 | -0.276500106 | Stam | P70297 | 3 | 3 | 3 |
| + | 2.042694511 | -0.329652399 | Polr1d | P97304 | 3 | 3 | 3 |
| + | 3.438593079 | -0.290620789 | Yrdc | Q3U5F4 | 3 | 3 | 3 |
| + | 2.729256843 | 0.487926006 | Sema4a | Q62178 | 3 | 3 | 3 |
| + | 1.726572598 | -0.686449289 | Lilrb4 | Q64281;Q61450 | 3 | 3 | 3 |
| + | 1.8414296 | -0.456112266 | Tsen2 | Q6P7W5 | 3 | 3 | 3 |
| + | 2.859571347 | -0.741624594 | Rhbdf2 | Q80WQ6 | 3 | 3 | 3 |
| + | 1.911610327 | -0.37876302 |  | Q8BGA7 | 3 | 3 | 3 |
| + | 2.542242431 | -0.334173322 | Pus7l | Q8CE46 | 3 | 3 | 3 |
| + | 3.065624304 | -0.36358659 | Stard7 | Q8R1R3 | 3 | 3 | 3 |
| + | 2.210338504 | -0.329427421 | Spg20 | Q8R1X6 | 3 | 3 | 3 |
| + | 2.170820808 | -0.356033027 | BC027231 | Q8R2U2 | 3 | 3 | 3 |
| + | 1.829042808 | 0.400012568 | Rabif | Q91X96 | 3 | 3 | 3 |
| + | 1.566455467 | -0.523700996 | Dst | Q91ZU6 | 7 | 3 | 3 |
| + | 1.951438834 | -0.345986873 | Npm3 | Q9CPP0 | 3 | 3 | 3 |
| + | 1.697977712 | -0.51154317 | Bcl2l14 | Q9CPT0 | 3 | 3 | 3 |
| + | 5.01859343 | -0.780247676 | Stx11 | Q9D3G5 | 3 | 3 | 3 |
| + | 2.342625871 | -0.347414494 | Tube1 | Q9D6T1 | 3 | 3 | 3 |
| + | 2.093643512 | 0.33551687 | Znf414 | Q9DCK4 | 3 | 3 | 3 |
| + | 2.108925982 | 0.392942488 | Osbpl5 | Q9ER64 | 4 | 3 | 3 |
| + | 2.591476847 | -0.310727386 | Ccdc86 | Q9JJ89 | 3 | 3 | 3 |
| + | 2.900746722 | -0.340830028 | Pole3 | Q9JKP7 | 3 | 3 | 3 |
| + | 3.157507135 | -0.538985536 | Tmem59 | Q9QY73 | 3 | 3 | 3 |
| + | 1.978133996 | 0.345723273 | Bak1 | O08734 | 4 | 4 | 4 |
| + | 2.826259015 | -0.545188665 | Pmm1 | O35621 | 6 | 4 | 4 |
| + | 1.613922707 | 0.834086433 | Fv4 | P11370 | 8 | 4 | 4 |
| + | 2.831803489 | -0.324110702 | Hmgn1 | P18608 | 4 | 4 | 4 |
| + | 2.142306682 | -0.505713671 | Eno3 | P21550 | 7 | 5 | 4 |
| + | 2.538318684 | -0.722266838 | Marcks | P26645 | 4 | 4 | 4 |
| + | 2.091925432 | -0.400150649 | Gla | P51569 | 4 | 4 | 4 |
| + | 3.838775529 | 1.216590283 | Ctse | P70269 | 4 | 4 | 4 |
| + | 1.541813949 | -0.467796295 | Wdr70 | Q3TWF6 | 4 | 4 | 4 |
| + | 2.001967213 | 0.388303697 | Tiam1 | Q60610 | 4 | 4 | 4 |
| + | 2.935509208 | 0.43810311 | Adat2 | Q6P6J0 | 4 | 4 | 4 |
| + | 3.45183989 | -0.266972452 | Hn1l | Q6PGH2 | 6 | 6 | 4 |
| + | 2.17601971 | -0.425465494 |  | Q8BL95 | 4 | 4 | 4 |
| + | 2.029565933 | -0.622988209 | Cenpu | Q8C4M7 | 4 | 4 | 4 |
| + | 2.741547079 | 0.468262494 | Dhx58 | Q99J87 | 4 | 4 | 4 |
| + | 5.666811777 | -1.523253202 |  | Q9CWU4 | 4 | 4 | 4 |
| + | 2.391852245 | 0.393582523 | Plgrkt | Q9D3P8 | 4 | 4 | 4 |
| + | 2.366564208 | -0.499764323 | Il21r | Q9JHX3 | 4 | 4 | 4 |
| + | 2.11818076 | 0.426763684 | Rab37 | Q9JKM7 | 4 | 4 | 4 |
| + | 2.491776539 | -0.312374592 | Tmf1 | B9EKI3 | 5 | 5 | 5 |
| + | 2.511379722 | -0.29668054 | Rpp30 | O88796 | 5 | 5 | 5 |
| + | 3.321910722 | -0.376965657 | Il4r | P16382 | 5 | 5 | 5 |
| + | 2.766111273 | -0.388007604 | Tnfrsf4 | P47741 | 5 | 5 | 5 |
| + | 1.970999982 | 0.368405245 | Ube2h | P62257 | 5 | 5 | 5 |
| + | 1.818887074 | -0.40590741 | Arid5a | Q3U108 | 5 | 5 | 5 |
| + | 1.701401492 | 0.431823574 | Ndufb6 | Q3UIU2 | 5 | 5 | 5 |
| + | 7.363504431 | -2.343394727 | Nnt | Q61941 | 5 | 5 | 5 |
| + | 1.428399966 | 0.527344257 | Mon1b | Q8BMQ8 | 5 | 5 | 5 |
| + | 2.534386724 | -0.545853786 | Cth | Q8VCN5 | 5 | 5 | 5 |
| + | 2.646289014 | 0.381955624 | Brap | Q99MP8 | 5 | 5 | 5 |
| + | 2.477641245 | 0.382501006 | Ndufb9 | Q9CQJ8 | 5 | 5 | 5 |
| + | 2.223217519 | -0.320751719 | Znf593 | Q9DB42 | 5 | 5 | 5 |
| + | 1.541252043 | -0.533354837 | Tnfrsf18 | O35714 | 6 | 6 | 6 |
| + | 3.249881712 | -0.545060946 | Bnip3 | O55003 | 6 | 6 | 6 |
| + | 2.609022119 | -0.306610404 | Surf2 | P09926 | 6 | 6 | 6 |
| + | 2.350661025 | -0.340972926 | Wbp4 | Q61048 | 6 | 6 | 6 |
| + | 3.131198956 | -0.276866704 |  | Q8C5K5 | 6 | 6 | 6 |
| + | 1.731405008 | -0.500635154 | Bag2 | Q91YN9 | 6 | 6 | 6 |
| + | 1.687261686 | -0.403790981 | Ehbp1l1 | Q99MS7 | 6 | 6 | 6 |
| + | 1.968300704 | -0.39356111 | Ybx3 | Q9JKB3;Q9Z2C8 | 8 | 6 | 6 |
| + | 2.612421141 | 0.307534745 | Lipa | Q9Z0M5 | 6 | 6 | 6 |
| + | 2.770431783 | -0.291342825 | Psmb5 | O55234 | 7 | 7 | 7 |
| + | 2.343819979 | -0.507413328 | Il2ra | P01590 | 7 | 7 | 7 |
| + | 2.145210471 | 0.523308225 | Lgals3 | P16110 | 7 | 7 | 7 |
| + | 3.841489268 | 0.236817158 | Qrich1 | Q3UA37 | 7 | 7 | 7 |
| + | 3.231959682 | 0.246367335 | Vps39 | Q8R5L3 | 7 | 7 | 7 |
| + | 2.415205162 | 0.310080737 | Rab27a | Q9ERI2 | 8 | 8 | 7 |
| + | 2.816584975 | -0.302458107 | Aprt | P08030 | 8 | 8 | 8 |
| + | 3.078429055 | -0.340731335 | Ldhb | P16125 | 9 | 8 | 8 |
| + | 1.824766936 | -0.528635103 | Nefh | P19246 | 10 | 8 | 8 |
| + | 2.243415069 | -0.346373585 | Il2rg | P34902 | 8 | 8 | 8 |
| + | 1.66301877 | -0.494162905 | Dnajb4 | Q9D832 | 9 | 8 | 8 |
| + | 2.918333023 | -0.6694184 | Gatm | Q9D964 | 8 | 8 | 8 |
| + | 1.692954625 | 0.521264829 | Capg | P24452 | 9 | 9 | 9 |
| + | 2.308651475 | -0.302617739 | Eci1 | P42125 | 9 | 9 | 9 |
| + | 1.965856231 | -0.353691876 | Cnbp | P53996;Q9D548 | 9 | 9 | 9 |
| + | 1.873131634 | -0.367537975 | Ybx1 | P62960 | 11 | 11 | 9 |
| + | 2.267401626 | -0.322993457 | Btf3 | Q64152 | 11 | 11 | 9 |
| + | 1.482012692 | -0.512240201 | Nucks1 | Q80XU3 | 9 | 9 | 9 |
| + | 2.530192415 | -0.433008835 | Sfr1 | Q8BP27 | 9 | 9 | 9 |
| + | 2.758441613 | 0.264190072 | Ttc39b | Q8BYY4 | 9 | 9 | 9 |
| + | 2.392500877 | -0.360151917 | Srfbp1 | Q9CZ91 | 9 | 9 | 9 |
| + | 2.175830763 | -0.689360525 | Cep192 | E9Q4Y4 | 10 | 10 | 10 |
| + | 2.604117672 | -0.312462032 | Eef1b | O70251 | 12 | 10 | 10 |
| + | 2.245538809 | -0.306390289 | Bcat1 | P24288 | 10 | 10 | 10 |
| + | 1.811113801 | -0.390772752 | Rrp1 | P56183 | 10 | 10 | 10 |
| + | 3.510277601 | 0.380059332 | Cd5 | P13379 | 11 | 11 | 11 |
| + | 3.752061969 | 0.26300849 | Cd6 | Q61003 | 13 | 13 | 13 |
| + | 1.855990035 | -0.440482095 | Slc4a7 | Q8BTY2 | 14 | 14 | 13 |
| + | 1.779514507 | -0.394623369 | Metap2 | O08663 | 15 | 15 | 15 |
| + | 2.349061705 | 0.481380105 | Gbp4 | Q61107 | 18 | 15 | 15 |
| + | 1.402724014 | 0.542721093 | Hist1h1a | P43275 | 19 | 16 | 16 |
| + | 1.778399156 | -0.533547074 | Irf4 | Q64287 | 16 | 16 | 16 |
| + | 2.621912858 | -0.289486498 | Mtdh | Q80WJ7 | 16 | 16 | 16 |
| + | 2.369475635 | -0.555456161 | Rap1gds1 | E9Q912 | 17 | 17 | 17 |
| + | 3.777285394 | 0.23122564 | Ctcf | Q61164;A2APF3 | 17 | 17 | 17 |
| + | 2.495483666 | 0.375007212 | Me2 | Q99KE1;Q8BMF3 | 19 | 19 | 19 |
| + | 3.210091364 | 0.462498426 | Hist1h4a | P62806 | 20 | 20 | 20 |
| + | 2.442901307 | -0.333532035 | Gnl3 | Q8CI11;REV__Q8BMK4;REV__E9PZM4;Q5SPX1 | 21 | 21 | 21 |
| + | 2.397455913 | -1.113331556 | Tgm2 | P21981 | 22 | 22 | 22 |
| + | 2.035293848 | 0.37149781 | Anxa11 | P97384 | 22 | 22 | 22 |
| + | 1.928775372 | -0.37620014 | Tax1bp1 | Q3UKC1 | 22 | 22 | 22 |
| + | 1.36045051 | -0.609189987 | Gbp2 | Q9Z0E6 | 27 | 22 | 22 |
| + | 3.069008715 | 1.5691939 | Gbp1 | Q01514 | 28 | 28 | 23 |
| + | 2.169269528 | 0.361834049 | Hp1bp3 | Q3TEA8 | 31 | 31 | 31 |
| + | 8.704709239 | 0.75314039 | Ide | Q9JHR7 | 32 | 32 | 32 |
| + | 4.181318913 | 0.328244805 | Mthfd1l | Q3V3R1 | 42 | 41 | 41 |
| + | 2.770683471 | 0.327286601 | Lmnb1 | P14733 | 48 | 48 | 42 |
| + | 2.656716397 | 0.545753241 | Ahnak | E9Q616;REV__P56375 | 199 | 199 | 199 |
