# Supplementary figures and images for: Patients and mice with deficiency in the SNARE protein SYNTAXIN-11 have a secondary B cell defect
Source: J Exp Med. 2024 May 9;221(7):e20221122. doi: 10.1084/jem.20221122 (PMC11082451; doi:10.1084/jem.20221122)

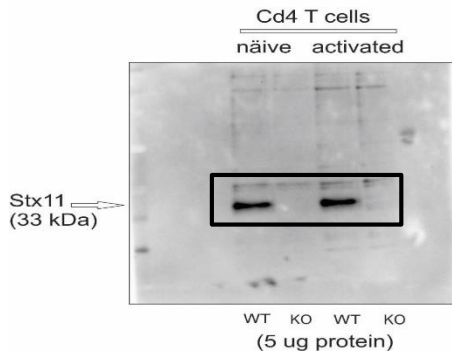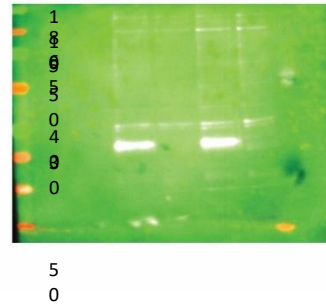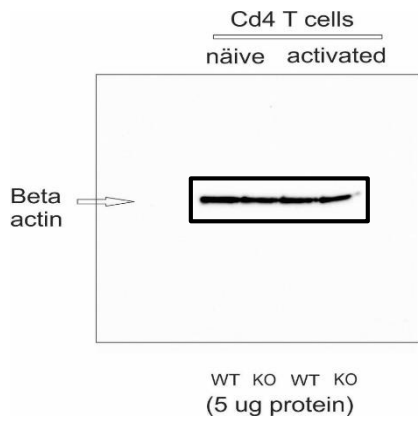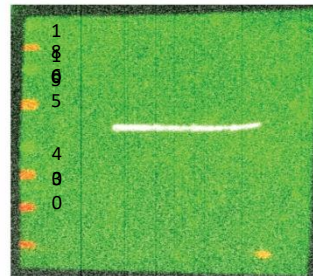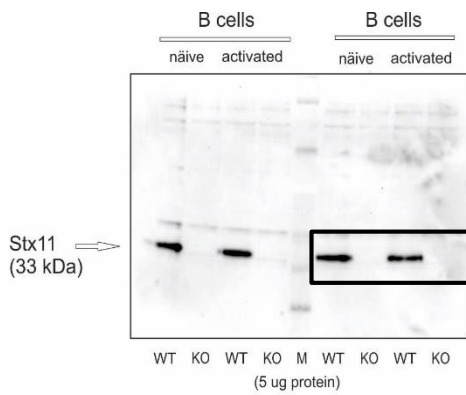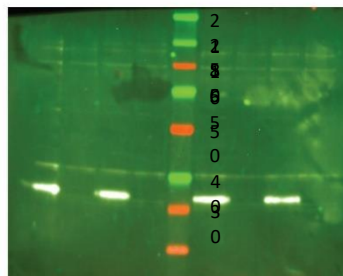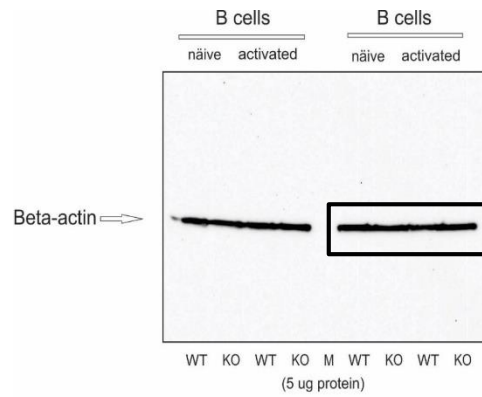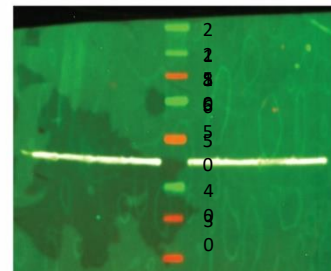

Supplement: SourceData FS2 — is the source file for Fig. S2. [file JEM_20221122_SourceDataFS2.pdf]

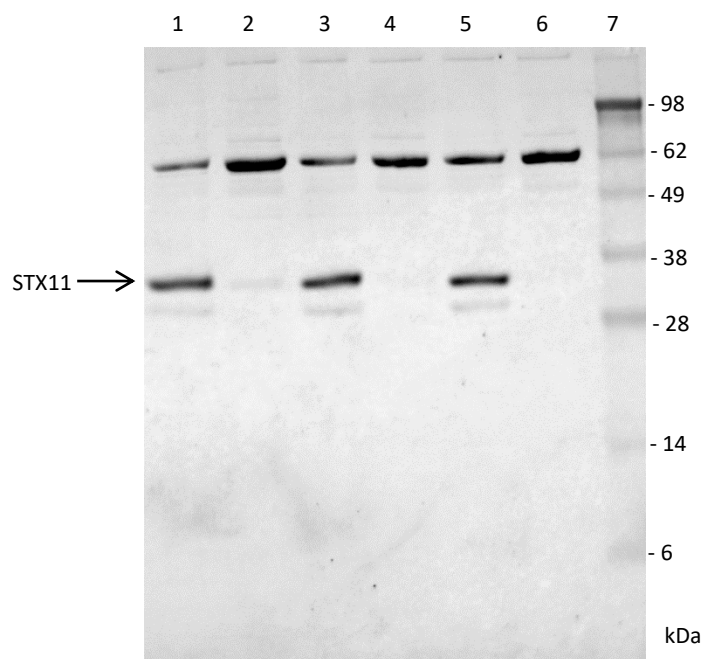

- 1 - UT 1
- 2 - KO 1
- 3 - UT 2
- 4 - KO 2
- 5 - UT 3
- 6 - KO 3
- 7 - Marker Seeblue Plus2

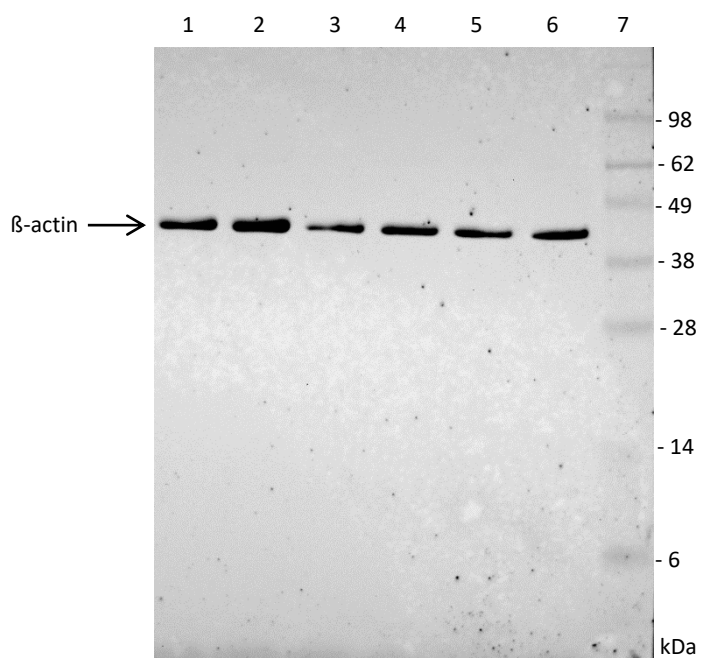

Supplement: SourceData F7 — is the source file for Fig. 7. [file JEM_20221122_SourceDataF7.pdf]
